# Solvatochromic covalent organic frameworks

**DOI:** 10.1038/s41467-018-06161-w

**Published:** 2018-09-18

**Authors:** Laura Ascherl, Emrys W. Evans, Matthias Hennemann, Daniele Di Nuzzo, Alexander G. Hufnagel, Michael Beetz, Richard H. Friend, Timothy Clark, Thomas Bein, Florian Auras

**Affiliations:** 1 Department of Chemistry and Center for NanoScience (CeNS), University of Munich (LMU), Butenandtstraße 5-13, 81377 Munich, Germany; 2 Cavendish Laboratory, University of Cambridge, Cambridge, CB3 0HE UK; 3 Computer-Chemie-Centrum, Department of Chemistry and Pharmacy, Friedrich-Alexander-University Erlangen-Nürnberg (FAU), Nägelsbachstraße 25, 91052 Erlangen, Germany

## Abstract

Covalent organic frameworks (COFs) are an emerging class of highly tuneable crystalline, porous materials. Here we report the first COFs that change their electronic structure reversibly depending on the surrounding atmosphere. These COFs can act as solid-state supramolecular solvatochromic sensors that show a strong colour change when exposed to humidity or solvent vapours, dependent on vapour concentration and solvent polarity. The excellent accessibility of the pores in vertically oriented films results in ultrafast response times below 200 ms, outperforming commercially available humidity sensors by more than an order of magnitude. Employing a solvatochromic COF film as a vapour-sensitive light filter, we demonstrate a fast humidity sensor with full reversibility and stability over at least 4000 cycles. Considering their immense chemical diversity and modular design, COFs with fine-tuned solvatochromic properties could broaden the range of possible applications for these materials in sensing and optoelectronics.

## Abstract (web summary)

Covalent organic frameworks (COFs) find increasing application as sensor material, but fast switching solvatochromism was not realized. Here the authors demonstrate that combination of electron-rich and -deficient building blocks leads to COFs which fast and reversibly change of their electronic structure depending on the surrounding atmosphere.

## Introduction

With covalent organic frameworks (COFs) already being in their early teens, the scientific community has gained a profound understanding regarding the synthesis of highly porous, crystalline and stable frameworks^1,2^. If these materials are to evolve from a purely academic research field, however, one of the next major challenges will be realizing COFs that can compete with established materials in practical applications.

COFs are formed via reversible cross-linking of rigid organic building blocks, whereby boronate esters^3–6^, imines^7–10^ and hydrazones^11,12^ represent the most prominent linkage motifs. Potential for application has mainly been demonstrated in the fields of gas storage^13–15^, catalysis and photocatalysis^16,17^, and in electronics and optoelectronics^18–22^. However, functionality arising from the combination of the well-defined porosity and the semiconducting properties of the COF backbone is still under-explored.

Taking particular advantage of their tuneable porosity and the resulting capability of selectively hosting specific guest molecules, a predestined ambit for COFs could be the sensing of ions or molecules. The COF-based sensing materials reported thus far are able to detect heavy metal ions^23,24^, pH changes^26^ or organic explosives^26,27^ via fluorescence quenching. A more general scope for application, however, would be the detection of water and solvent vapours in the gas phase with the possibility of differentiating between various substances. COFs featuring this kind of nosing capability could be a powerful tool for detecting harmful volatile organic compounds in workplace environments, or for real-time monitoring of the water content of gas and solvent streams in industrial processes. Such on-line analysis would require an easy read-out possibility, preferably via a colour change of the detector material, in combination with full reversibility over multiple cycles and sufficient photochemical stability.

Reversible colour changes of solvated organic molecules as a function of the solvent polarity are known as solvatochromism. This effect occurs when the ground and excited states of a molecule are of different polarity, thus rendering the energy of intramolecular electronic transitions sensitive to changes in the polarity of the surrounding medium^29^. Charge-transfer transitions, as they occur in the archetypic Reichardt’s dye (2,6-diphenyl-4-(2,4,6-triphenylpyridinium)phenolate, betaine 30), display the highest sensitivity in this context^30^.

The solvatochromic effect has mostly been exploited for defining solvent polarity scales such as the *E*_T_(30) and the normalized *E*_T_^N^ scales through measuring solvent-dependent energy shifts of the absorption onset^31^. For detecting target molecules in a stream of gas or liquid, however, a dissolved molecular dye would be highly impractical.

A suitably designed COF, on the other hand, could constitute a supramolecular periodic analogue of the aforementioned solvatochromic dyes, with the added benefit of being an insoluble, chemically and photochemically stable material^31,32^. The modular COF design allows for matched combinations of electron-rich and -deficient building blocks, generating a periodic lattice of covalently linked donor–acceptor pairs that promote charge-transfer transitions^33,34^. For optimal performance and fast response times, COFs can be grown as thin films with their pores oriented vertically to the substrate, thus exposing their high internal surface area to the analyte^35,36^.

Here we present oriented thin films of tetrakis(4-aminophenyl)pyrene-based COFs that show an ultrafast and fully reversible solvatochromic response upon exposure to various polar and non-polar vapours. The newly developed COFs derive their high degree of crystallinity from geometric interlocking of the covalently linked two-dimensional (2D) sheets into a synchronized offset-stacked pattern^10,37^. The charge-transfer character of the optical transitions and hence the sensitivity to changes in polarity inside the COF pores can be tuned through the aldehyde counterpart used for assembling the COF. In this context, the strong donor–acceptor contrast realized between tetraphenylpyrene and thieno[3,2-*b*]thiophene yields the most pronounced solvatochromism. Oriented thin films of this COF with the pores extending vertically from the substrate exhibit millisecond response times to changes in the surrounding atmosphere and fully retain their structure and function over several thousand cycles.

## Results

### COF design

We selected the electron-rich 1,3,6,8-tetrakis(4-aminophenyl)pyrene, Py(NH_2_)_4_, as a basis for constructing solvatochromic COFs. Pyrene-based COFs have not only proven to yield extremely well-ordered frameworks with large crystal domains in our recent studies^37^, but are also geometrically compatible with a wide range of aromatic and heteroaromatic aldehyde counterparts^33,34,38^, enabling us to optimize the solvatochromic response within a single COF family.

Combinations with more electron-deficient aldehyde counterparts are expected to produce electronic transitions with a varying degree of charge-transfer character across the conjugated imine bond. For optimal solvatochromic response, however, these charge-transfer transitions are not only required to possess sufficient oscillator strength, but must also be sensitive to polarity changes in the pores.

**Fig. 1:**
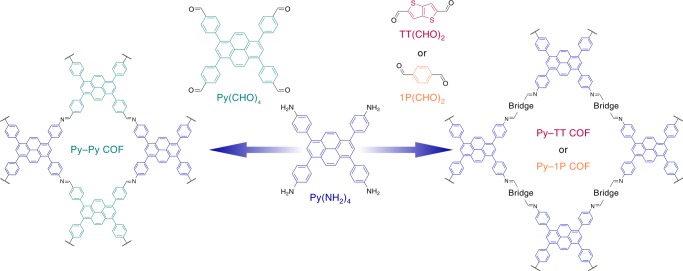
Synthesis of the imine-linked COFs. Combining the tetraphenylpyrene tetraamine Py(NH_2_)_4_ with the tetradentate pyrene aldehyde Py(CHO)_4_ in a 1:1 molar ratio yields the microporous Py–Py COF (left), whereas the combination of Py(NH_2_)_4_ with linear dialdehydes in a 1:2 molar ratio produces the mesoporous Py–TT and Py–1P COFs, respectively (right)

In view of these considerations, we chose three increasingly electron-deficient aldehyde counterparts (Fig. 1). Pairing Py(NH_2_)_4_ with the tetradentate 1,3,6,8-tetrakis(4-formylphenyl)pyrene, Py(CHO)_4_, in the Py–Py COF is anticipated to produce the smallest donor–acceptor contrast in this context, derived mainly from the slightly polarized, electron-accepting imine bond. Switching from pairing two tetradentate building blocks to a combination of the tetradentate amine with a linear acene dialdehyde, 1P(CHO)_2_, as realized in the Py–1P COF, increases the polarity within the linear bridge and doubles the number of weakly accepting imines. A much stronger charge-transfer character can be achieved in combinations with electron-deficient heterocycles, such as the thieno-[3,2-*b*]thiophene-2,5-dicarboxaldehyde, TT(CHO)_2_, in the Py–TT COF.

### COF bulk materials

The Py–Py, Py–1P and Py–TT COFs were initially synthesized as bulk powders under solvothermal conditions (see the Methods section and Supplementary Methods for details, and Supporting Figures, section Q for infrared spectra and thermogravimetric analysis).

**Fig. 2:**
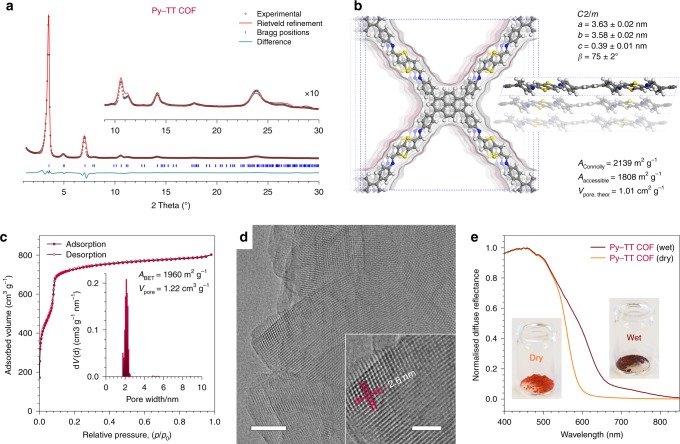
Structure analysis and solvatochromism of the Py–TT COF bulk powder. **a** Experimental PXRD pattern (black dots) of the Py–TT COF powder. Rietveld refinement (red line) using the structure model displayed in **b** provides a very good fit to the experimental data with only minimal differences between the experimental and the refined patterns (green line). *R*_wp_ = 4.9%, *R*_p_ = 10.5%. Bragg positions are indicated by blue ticks. Inset, magnified view of the 2θ > 9° region. **b** Top view (left) and side view (right) of the corresponding unit cell reveal the pseudo-quadratic, offset-stacked structure that is typical for pyrene-based COFs. Crystallographic data are available as Supplementary Data 1. The structure has a Connolly surface of 2139 m^2^ g^−1^, an accessible surface area of 1808 m^2^ g^−1^ and a pore volume of 1.01 cm^3^ g^−1^. **c** Nitrogen sorption isotherm of the Py–TT COF recorded at 77 K. Inset, QSDFT calculation using an equilibrium model yields a very narrow pore size distribution with a maximum at 2.1 nm. **d** High-resolution TEM image showing the large crystal domains of the Py–TT COF. Scale bar: 40 nm. Inset, magnified view onto a COF crystallite visualizing the pseudo-quadratic arrangement of the COF pores with a periodicity of 2.6 ± 0.1 nm. Scale bar: 20 nm. **e** Diffuse reflectance spectra of the dry (orange) and water vapour-saturated (brown) Py–TT COF powder showing a strong solvatochromic red-shift

The powder X-ray diffraction (PXRD) pattern of the Py–TT COF contains a number of sharp reflections, including several well-defined higher-order reflections, and is devoid of any visible amorphous background (Fig. 2a). Rietveld refinement employing the density functional theory (DFT)-optimized *C*2/*m*-symmetric structure model shown in Fig. 2b (see the Supplementary Methods for details) provides a very good fit to the experimental data. However, the large number of light atoms in the unit cell and peak broadening due to the inherent flexibility of imine-linked COFs impede the refinement of individual atom positions. Hence, slight differences between the structure model and the actual COF structure can cause the deviations in intensity that we observe for some of the higher-index reflections.

With pore diagonals of 2.4 and 2.0 nm (corner-to-corner and bridge-to-bridge, respectively) in the refined structure model, the Py–TT COF is expected to be a mesoporous material. Its nitrogen sorption isotherm exhibits a type IVb isotherm shape with a sharp step at *p*/*p*_0_ = 0.08, confirming the mesoporosity (Fig. 2c)^40^. Quenched solid density functional theory (QSDFT) analysis using an equilibrium model for cylindrical pores yields a very narrow pore size distribution with a maximum of 2.1 nm, in excellent agreement with the structure model. The Brunauer–Emmett–Teller (BET) surface of the Py–TT COF is 1960 ± 50 m^2^ g^−1^ with a total pore volume of 1.22 ± 0.05 cm^3^ g^−1^. These results are in very good agreement with the porosity values derived from the structure model, confirming that the pores of the framework are fully open and accessible.

Transmission electron microscopy (TEM) reveals the formation of a periodic framework with domain sizes of 50–200 nm (Fig. 2d). High-resolution TEM confirms the pseudo-quadratic geometry of the COF with a periodicity of 2.6 ± 0.1 nm, in excellent agreement with the pore-to-pore repeat distance of 2.5 nm in the refined structure model.

The isostructural Py–1P COF is an equally well-crystallized framework with a slightly smaller unit cell due to the shorter terephthalaldehyde bridge (Supplementary Figure 1). The Py–Py COF has a similar pseudo-quadratic overall geometry, but is composed of alternating columns of the pyrene amine and aldehyde. The symmetry of the framework is thus reduced to *P*2/*m* with a considerably smaller unit cell owing to the reduced length of the bridge between the pyrene centres (Supplementary Figure 2).

Following our initial considerations, COFs comprising donor–acceptor motifs of alternating electron-rich and -deficient building blocks are expected to show a solvatochromic response towards molecules in their pores. Indeed, exposing the initially orange-red Py–TT COF powder to an atmosphere of 98% relative humidity causes a colour change to dark brown within a few seconds (Fig. 2e). The corresponding diffuse reflectance spectra reveal that this colour change stems from the appearance of new optical transitions in the 550–850 nm range. This effect is fully reversible as the colour reverts to the initial orange-red hue upon drying. The Py–1P and Py–Py COFs also respond to water vapour (Supplementary Figures 1e and 2e). The colour shifts, however, are less pronounced, presumably owing to the much smaller donor–acceptor contrast between their building blocks. The origin of the solvatochromic colour shifts in our COFs will be discussed in more detail below.

### COF thin films

We anticipated that growing the solvatochromic COF as an oriented film with the pores extending from the substrate surface would greatly facilitate the diffusion of guest molecules into and out of the framework and thus strongly accelerate the response to changes in the surrounding atmosphere. Supported COF thin films would moreover simplify handling, improve re-usability and facilitate the read-out procedure in sensing applications (see below).

The growth of oriented COF films on non-epitaxial substrates has recently also been realized for imine-linked frameworks^35^. We adapted this method for the growth of the Py–TT, Py–1P and Py–Py COFs. Solvothermal syntheses in slightly diluted solutions yielded smooth and homogeneous films of the three frameworks on fused silica, sapphire or indium-tin-oxide (ITO) substrates with tuneable thickness between 160 and 360 nm, depending on the reaction time (see the Methods section and Supplementary Methods for details).

**Fig. 3:**
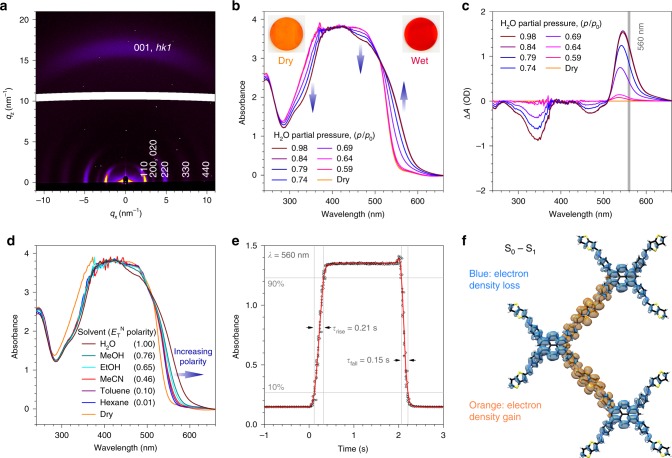
Solvatochromism of Py–TT COF oriented thin films. **a** GIWAXS pattern of a 360 nm thick Py–TT COF film grown on a sapphire substrate. The intensity of *hk*0 reflections is concentrated directly above the sample horizon, whereas the 001 and other low-index *hk*1 reflections appear close to the substrate normal. **b** UV–Vis absorption spectra of the Py–TT COF film recorded at different relative pressures of H_2_O in N_2_. Increasing water content causes a strong absorption increase in the 520–640 nm region, accompanied by a reduced absorption in the 440–500 nm and 280–380 nm regions. Insets, photographs of the COF film in the dry and water-saturated states. **c** Corresponding plot of the humidity-induced absorbance changes, *A*_humid_−*A*_dry_, at different H_2_O relative pressures. The grey line indicates the wavelength used for the response time measurements (see below). **d** UV–Vis spectra of the same COF film in saturated atmospheres of various solvents. The solvatochromic shift increases monotonically with increasing *E*_T_^N^ polarity of the solvents. **e** Solvatochromic response of the Py–TT COF film towards step changes between dry and H_2_O-saturated N_2_ streams. Ten individual data sets (black dots) recorded at *λ* = 560 nm have been averaged (red line). The response times *τ*_rise_ and *τ*_fall_ are determined between the 10% and 90% thresholds. **f** TD-DFT calculated electron density difference upon the one-electron excitation from the ground state (*S*_0_) to the first singlet excited state (*S*_1_). This transition involves a reduction of the electron density on the pyrene moieties and an electron density gain of the thienothiophene bridges, and hence possesses a pronounced charge-transfer character. Electron density isosurfaces are displayed at an isovalue of 5e−5

The 2D grazing-incidence wide-angle X-ray scattering (GIWAXS) pattern of the Py–TT COF film exhibits a number of well-defined reflections that can be indexed as shown in Fig. 3a. The distribution of the reflections indicates that the COF film is highly textured with the imine-linked layers extending parallel to the substrate surface (Supplementary Figure 3a, b). Individual COF domains grow hereby at random rotation about the substrate normal (planar disorder) (Supplementary Figure 3c, d). We found that this texture is identical for different substrates (*c*-cut sapphire, fused silica, polycrystalline ITO), suggesting that the uniaxial preferred orientation is generated by the anisotropy of the framework^34,36^.

The electronic coupling throughout the film was analysed for films grown on ITO substrates with electron- or hole-selective contact layers (TiO_2_ or MoO_*x*_, respectively). Transport measurements of these vertical-stack single-carrier devices yield charge-carrier mobilities of (4.02 ± 0.04) × 10^−6^ and (1.02 ± 0.01) × 10^−7^ cm^2^ V^−1^ s^−1^ for holes and electrons, respectively (see Supplementary Figures, section G).

In accordance with the GIWAXS results, TEM analysis of a Py–TT COF film removed from the substrate shows a highly textured morphology with the *ab*-plane perpendicular to the viewing direction, i.e., parallel to the substrate (Supplementary Figure 3e, f). The pores consequently extend at an angle of 75° vs. the substrate surface (i.e., 15° vs. the viewing direction) and hence are fully accessible to the surrounding atmosphere.

For the characterization of the Py–1P and Py–Py COF thin films, see Supplementary Figure 11.

### Solvatochromism

As in the case of the COF powder, exposing the Py–TT COF film to a humidified N_2_ stream results in a colour change from orange to dark red. Transmission ultraviolet–visible (UV–Vis) spectra recorded at different H_2_O relative pressures reveal the appearance of an absorption band in the 520–640 nm region and a simultaneous decrease in absorption across the 440–500 nm and 280–380 nm spectral regions (Fig. 3b). Plotting the change in absorbance ∆*A* enables us to identify a strong humidity-induced absorption band with a maximum at 545 nm that is accompanied by two bleach bands with minima at 345 and 470 nm (Fig. 3c). The COF film exhibits the highest sensitivity towards humidity changes between H_2_O relative pressures of 0.64 and 0.79. Above this, the absorption change saturates, possibly due to condensation in the COF pores.

The Py–TT COF was found to respond in a similar way to a range of organic solvents (Fig. 3d). The magnitude of the colour change upon exposure to a saturated atmosphere increases hereby monotonically with the *E*_T_^N^ polarity of the respective solvent (Supplementary Figure 5)^31^. The Py–TT COF thus represents a solid-state supramolecular analogue to the commercially available molecular solvatochromic dyes with the added benefit of being sensitive even to vapours diluted in a carrier gas.

Oriented films of the Py–TT COF display a very fast response towards step changes between dry and H_2_O-saturated gas streams (Fig. 3e). For a 360 nm thick film, we observe a response time (*τ*_rise_) of 0.21 s for the absorption increase upon change from a dry to humid atmosphere, while the transition to the dry state is even faster with *τ*_fall_ = 0.15 s. As the solvent molecules need to diffuse through the entire film in order to saturate the solvatochromic colour change, we anticipated a strong correlation between film thickness and response time. Indeed, both response times get shorter for thinner films, whereby the fastest response of 0.11 s/0.09 s (rise/fall) was achieved using a 160 nm thick COF film (Supplementary Figure 6). To the best of our knowledge, this represents the fastest response time of a solvatochromic sensing system reported to date, and places it among the fastest nanostructured humidity sensors^40–43^.

In addition to this extremely fast response, the Py–TT COF films display excellent reversibility and reproducibility during repeated switching (Supplementary Figure 7a). Furthermore, the COF film is stable over at least 4000 humidity and solvent vapour switching cycles and storage in ambient air for 250 days, without showing any apparent changes in its absorption spectra and GIWAXS patterns (Supplementary Figure 7b–e).

For a possible application as a high-performance solvatochromic sensor, easy read-out, fast response times, reversibility and reproducibility are of key importance. Employing the COF thin film as a vapour-sensitive light filter, a continuous read-out was realized in combination with a green light-emitting diode (LED) and a light-dependent resistor (Supplementary Figure 17). A video demonstrating this proof of concept is included as Supplementary Movie.

In order to clarify the origin of the solvent-induced colour changes of our COFs, we first need to exclude any chemical or structural changes that might alter the coupling between the building blocks or COF layers. Solid-state nuclear magnetic resonance measurements give no indication of a different chemical environment in the water-saturated COF (Supplementary Figure 10). If the Py–TT COF is exposed to humidity, the PXRD reflection intensities, especially of *hk*0 reflections, are reduced considerably (Supplementary Figure 9a, b). This effect, however, is fully reversible and can be attributed to modified structure factors due to the water molecules in the pores (Supplementary Figure 9c, d). All reflection positions, peak shapes and widths, and hence the unit cell and framework symmetry, remain unchanged during the humidity cycles. Given the three-dimensional configuration and interlocked stacking of the COF layers, even minor deformations or rotations of the bridges would be reflected in modified unit cell parameters. The absence of structural changes is further supported by the extreme stability of the material, which seems hardly possible if deformations or sliding of the COF layers were involved. Moreover, the as-synthesized COFs are, despite their acid-catalysed formation, not protonated (Supplementary Figure 12). Protonation of the imines, which is possible with strong acids, produces a different and more red-shifted absorption profile than the solvatochromism.

The Py–TT COF displays a positive solvatochromism, i.e., the absorption is red-shifted with increasing polarity. In well-studied molecular dyes such as phenol blue^45^, this is observed for combinations of a low-polarity ground state with a polar first excited state^29^. In that case, the excited state is stabilized to a greater extent than the ground state with increasing polarity of the surrounding medium, hence lowering the energy required for photoexcitation.

To take a closer look at the electronic structure of the COF, we performed time-dependent density functional theory (TD-DFT) calculations (PBE0/6-31G(d)) for a single-layer Py–TT molecular fragment (see the Supplementary Figures, section S for details). The electron density difference upon the one-electron excitation from the ground state to the first singlet excited state reveals that this transition involves a reduction of the electron density on the pyrene moieties, accompanied by an electron density gain of the thienothiophene bridges (Fig. 3f). Hence, this lowest-energy optical transition has significant charge-transfer character, as we anticipated from our initial considerations. The effect of a surrounding solvent was probed by employing the conductor-like polarizable continuum model^46^. In support of the experimental data, the calculated absorption in water is red-shifted with respect to the absorption in vacuum (705 nm vs. 685 nm). The absolute energies of the transitions are about 0.4 eV lower than the experimental values due to limitations of the TD-DFT method.

These findings are further supported by the photoluminescence (PL) characteristics of the Py–TT COF (Supplementary Figure 8). If the COF is exposed to a humid atmosphere, the PL is red-shifted, indicating a stabilization of the excited state by the pore medium. This is accompanied by a reduction in PL intensity by more than 95% compared to the dry material, suggesting that the increased dielectric screening due to the water molecules helps to overcome the Coulomb barrier and sustain a more charge-separated state^46,47^. This sensitivity to the brought-in charge-transfer character causes the solvatochromic colour shifts.

While the above findings provide strong evidence for a purely electronic nature of the solvatochromism itself with no structural or chemical changes involved, the morphology of our materials is crucial for obtaining observable colour shifts and fast response times. Thin films of a Py–1P molecular fragment and of an amorphous Py–1P network, despite being chemically and electronically almost identical to the crystalline Py–1P COF, do not show any measurable solvatochromism (see the Supplementary Figures, section P for details). Only the COF with its regular microporosity provides the required accessibility on a molecular length scale, allowing the water or solvent molecules to rapidly penetrate into the material and trigger the electronic changes.

## Discussion

We have developed the first solvatochromic covalent organic frameworks that show strong colour shifts when exposed to solvent or water vapours. Growing these COFs as highly crystalline vertically oriented thin films, we have realized optically homogeneous coatings that can act as fully reversible, solid-state supramolecular solvatochromic sensors. The excellent accessibility of the pores in these films results in ultrafast response times of below 200 ms, thus outperforming commercially available humidity sensors by more than an order of magnitude. As a proof of concept, we constructed a simple and fast humidity sensor device by using the COF film as a vapour-sensitive light filter between an LED and a light-dependent resistor. Experimental data and TD-DFT calculations strongly suggest that the solvatochromism is of purely electronic origin and does not involve structural or chemical changes in the framework – a fact that we believe is not only key to the extremely fast response times and outstanding stability of the material, but might also have implications for the use of COFs in the broader context of optoelectronics. In particular, the observation that in these materials electronic transitions can be manipulated reversibly and that intramolecular charge-transfer can be facilitated via the inclusion of chemically inert guest molecules could impact the development of stimuli-responsive organic electronics. Future chemical modifications to the COF backbone or the pore walls could be used to adapt the sensitivity and selectivity of the solvatochromic response, broadening the range of possible applications for these materials.

## Methods

### Py–TT COF synthesis

COF bulk powder syntheses were performed under argon atmosphere in polytetrafluoroethylene (PTFE)-sealed glass reaction tubes (6 mL volume). Solvents and acetic acid were obtained in high-purity grades from commercial suppliers and were, unless shipped under argon, degassed and saturated with argon prior to use.

Py(NH_2_)_4_ (14.0 mg, 20 µmol) and thieno-[3,2-*b*]thiophene-2,5-dicarboxaldehyde (7.8 mg, 40 µmol) were filled into a reaction tube, followed by the addition of mesitylene (667 µL), benzyl alcohol (333 µL) and 6 M acetic acid (100 µL). The tube was sealed and kept at 120 °C for 3 days. After cooling to room temperature, the precipitate was collected by filtration, washed with MeCN and dried in air, yielding a bright red powder.

### Py–TT COF thin film synthesis

COF thin films were synthesized in 100 mL autoclaves equipped with a 28 mm diameter glass liner. Fused silica (Spectrosil 2000), sapphire (UQG Optics, *c*-axis cut) and ITO-coated glass (VisionTec, 12–15 ohms per sq) substrates were cleaned in detergent solution, water, acetone and isopropanol, and activated with an O_2_-plasma for 5 min directly before use. The substrates were placed horizontally in PTFE sample holders with the activated surface face-down.

Py(NH_2_)_4_ (7.0 mg, 10 µmol) and thieno-[3,2-*b*]thiophene-2,5-dicarboxaldehyde (4.0 mg, 20 µmol) were filled into an autoclave, followed by the addition of mesitylene (1333 µL) and benzyl alcohol (666 µL). A substrate (fused silica, sapphire or ITO) was inserted, followed by the addition of 6 M acetic acid (200 µL). The autoclave was sealed and heated to 120 °C for 4 days. After cooling to room temperature, the substrate was immersed in dry MeCN and dried with compressed air. Thinner films were grown at shorter reaction times ranging from 4 h to 2 days.

### Structure characterization

PXRD measurements were performed using a Bruker D8 Discover with Ni-filtered Cu K_*α*_ radiation and a LynxEye position-sensitive detector.

The 2D GIWAXS data were recorded with an Anton Paar SAXSpace system equipped with a GeniX Cu K_*α*_ microsource and a Dectris Eiger R 1M detector. The samples were positioned at a tilt angle of 2.3° and a sample-detector distance of 135 mm.

TEM was performed with an FEI Titan Themis equipped with a field emission gun operated at 300 kV.

### Optical absorption spectroscopy

UV–Vis spectra were recorded using a Perkin-Elmer Lambda 1050 spectrometer equipped with a 150 mm InGaAs integrating sphere. Time-resolved absorption measurements were performed at fixed detector gain and slit settings. Diffuse reflectance spectra were collected with a Praying Mantis (Harrick) accessory and were referenced to barium sulphate powder as white standard. The specular reflection of the sample surface was removed from the signal by spatial filtering.

### Gas flow experiments

Gas flow experiments were performed using a gas flow controller system (F-201-C-RBA-33-V, Bronkhorst Hi-Tec) and a liquid mass flow controller with a controlled evaporation mixer (W-101A-110, Bronkhorst Hi-Tec), where the solvents were evaporated at temperatures above their boiling points. Solvents were obtained from commercial suppliers in high-purity anhydrous grades and were used as received. The flow cell was home-built from a 10 × 10 mm fused silica cuvette (Hellma Analytics) equipped with a tightly fitting PTFE lid and 2 mm diameter PP hoses connected to the gas flow system.

## Electronic supplementary material

Supplementary Information

Description of Additional Supplementary Files

Supplementary Movie 1

Supplementary Dataset 1

Supplementary Dataset 2

Supplementary Dataset 3

## Data Availability

The data that support the findings of this study are available within the article and supplementary information files, or available from the corresponding authors on reasonable request.
