# Supplementary material for: Solvatochromic covalent organic frameworks
Source: Nat Commun. 2018 Sep 18;9:3802. doi: 10.1038/s41467-018-06161-w (PMC6143592; doi:10.1038/s41467-018-06161-w)
Supplement: Supplementary file 1 — Supplementary Information [file 41467_2018_6161_MOESM1_ESM.pdf]

Supplementary Information

Solvatochromic covalent organic frameworks

Laura Ascherl[1], Emrys W. Evans[2], Matthias Hennemann[3], Daniele Di Nuzzo[2], Alexander G. Hufnagel[1], Michael Beetz[1], Richard H. Friend[2], Timothy Clark[3], Thomas Bein[1]* and Florian Auras[2]*

[1]Department of Chemistry and Center for for NanoScience (CeNS), University of Munich (LMU), Butenandtstraße 5-13, 81377 Munich, Germany

[2]Cavendish Laboratory, University of Cambridge, Cambridge CB3 0HE, United Kingdom

[3]Computer-Chemie-Centrum, Department of Chemistry and Pharmacy, Friedrich-Alexander-University Erlangen-Nürnberg (FAU), Nägelsbachstraße 25, 91052 Erlangen, Germany

*e-mail: bein@lmu.de, fa355@cam.ac.uk

Table of contents

Supplementary Methods

A Characterisation methods . . 2

B Building block syntheses . . 3

C COF syntheses . . . 4

Supplementary Figures

D Py-1P COF structure analysis . 5

E Py-Py COF structure analysis . 6

F Py-TT COF film morphology . 7

G Py-TT COF single carrier devices . 8

H Solvent-induced spectral changes . 9

I Thickness-dependent response times 9

J Reproducibility and stability . 10

K Py-TT COF photoluminescence spectra 10

L Humidity-dependent PXRD analysis . 11

M Solid-state NMR spectroscopy . 11

N Analysis of the Py-1P and Py-Py COF films 12

O Protonation experiments . . 13

P Py-1P fragments and networks . 14

Q Additional COF characterisation . 16

R Py-TT COF based humidity sensor . 17

S TD-DFT simulations . . 18

Supplementary References . . 20

Abbreviations

BET	Brunauer-Emmett-Teller
DCM	dichloromethane
DFT	density functional theory
DMF	*N*,*N*-dimethylformamide
DMSO	dimethyl sulfoxide
eq.	equivalents
GIWAXS	grazing-incidence wide angle X-ray scattering
ITO	indium tin oxide
LED	light-emitting diode
LDR	light-dependent resistor
NMR	nuclear magnetic resonance
PP	polypropylene
PTFE	poly(tetrafluoroethylene)
PXRD	powder X-ray diffraction
QSDFT	quenched solid density functional theory
RH	relative humidity
SCLC	space-charge-limited current
SEM	scanning electron microscopy
TD-DFT	time-dependent density functional theory
TEM	transmission electron microscopy

Supplementary Methods

A. Characterisation methods

Nuclear magnetic resonance (NMR) spectra were recorded on Bruker AV 400 and AV 400 TR spectrometers. Proton chemical shifts are expressed in parts per million (δ scale) and are calibrated using residual undeuterated solvent peaks as an internal reference (DMSO-d_6: 2.50). Data for ^{1}H NMR spectra are reported in the following way: chemical shift (δ ppm) (multiplicity, coupling constant, integration). Multiplicities are reported as follows: s = singlet, d = doublet, t = triplet, q = quartet, m = multiplet, or combinations thereof. Magic angle spinning (MAS) **solid-state nuclear magnetic resonance** (ssNMR) spectra were recorded with a Bruker Avance III-500 spectrometer using 2.5 mm diameter ZrO_2 rotors at a spinning frequency of 20 kHz.

Powder X-ray diffraction (PXRD) measurements were performed using a Bruker D8 Discover with Ni-filtered Cu K_α radiation and a LynxEye position-sensitive detector.

2D **grazing-incidence wide angle X-ray scattering** (GIWAXS) data were recorded with an Anton Paar SAXSpace system equipped with a GeniX Cu K_α microsource and a Dectris Eiger R 1M detector. The samples were positioned at a tilt angle of 2.3° and a sample-detector distance of 135 mm.

The **structure models of the COFs** were constructed using the Accelrys Materials Studio software package. For each COF structure we applied the space group with the highest possible symmetry, taking into account the rotation of the phenylenes versus the pyrene core. **DFT-based geometry optimisations** were performed with the CASTEP code using the generalized gradient approximation PBE functional.[1,2] The energy cutoff for the plane-wave basis set was set to 310.0 eV, ions were represented with ultrasoft pseudopotentials and k-point sampling was performed with a 1x1x4 Monkhorst-Pack grid.[3] The correction scheme of Tkatchenko and Scheffler was used to account for dispersion interactions.[4] **Structure refinements** using the Rietveld method were carried out as implemented in the Reflex module of the Materials Studio software. Pseudo-Voigt peak profiles were used and peak asymmetry was corrected using the Finger-Cox-Jephcoat method. **Connolly surfaces** and accessible surfaces were generated using an N_2-sized probe (r = 0.184 nm) at a 0.025 nm grid interval.[5]

Nitrogen sorption isotherms were recorded on a Quantachrome Autosorb 1 at 77 K. Prior to the measurements, the samples were outgassed for 24 h at 120°C under high vacuum. For the evaluation of the surface areas the BET model was applied in the ranges $0.005 \leq p/p_0 \leq 0.025$ (Py-TT COF), $0.002 \leq p/p_0 \leq 0.023$ (Py-1P COF), and $0.0002 \leq p/p_0 \leq 0.004$ (Py-Py COF), respectively. Total pore volumes were determined at $p/p_0 = 0.9$ to minimize the contributions of textural porosity. Pore size distributions were calculated using the QSDFT equilibrium model with a carbon kernel for cylindrical pores.

Transmission electron microscopy (TEM) was performed with an FEI Titan Themis equipped with a field emission gun operated at 300 kV.

Scanning electron microscopy (SEM) was performed with an FEI Helios NanoLab G3 UC eqiupped with a Schottky field-emission electron source operated at 1 - 30 kV.

UV-Vis spectra were recorded using a Perkin-Elmer Lambda 1050 spectrometer equipped with a 150 mm InGaAs integrating sphere. Time-resolved absorption measurements were performed at fixed detector gain and slit settings. **Diffuse reflectance spectra** were collected with a Praying Mantis (Harrick) accessory and were referenced to barium sulfate powder as white standard. The specular reflection of the sample surface was removed from the signal by spatial filtering.

Gas flow experiments were performed using a gas flow controller system (F-201-C-RBA-33-V, Bronkhorst Hi-Tec) and a liquid mass flow controller with a controlled evaporation mixer (W-101A-110, Bronkhorst Hi-Tec), where the solvents were evaporated at temperatures above their boiling points. Solvents were obtained from commercial suppliers in high-purity anhydrous grades and were used as received. The flow cell was home-built from a 10 x 10 mm fused silica cuvette (Hellma Analytics) equipped with a tightly fitting PTFE lid and 2 mm diameter PP hoses connected to the gas flow system. Individual vapour pressures were calculated using the Bronkhorst Fluidat software tool.[6] In this context the terms "dry" and "wet" refer to H_2O partial vapour pressures of $p/p_0 = 0$ and $p/p_0 = 0.98$, respectively.

Steady-state **humidification** of the bulk powders was performed using a home-built humidification chamber with a saturated potassium sulfate solution to reach a relative humidity of 98%.[7]

Photoluminescence (PL) measurements were performed using a home-built setup consisting of a Horiba Jobin Yvon iHR 320 monochromator equipped with a photomultiplier tube and a liquid N_2-cooled InGaAsdetector. The samples were illuminated with a 378 nm diode laser (pulse power 0.99 nJ cm^{-2}, pulse rate 40 MHz).

FT-IR spectra were recorded using a Perkin Elmer Spectrum BX FT-IR System.

B. Building block syntheses

All reactions were performed in oven-dried glassware under argon atmosphere using standard Schlenk and glovebox techniques. Commercially available reagents were used as received. Solvents were obtained in high-purity grades from commercial suppliers and were, unless shipped under argon, degassed and saturated with argon prior to use.

1,3,6,8-tetrakis(4-aminophenyl)pyrene, Py(NH$_2$)$_4$[8,9]

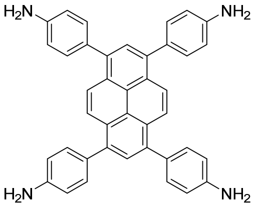

A reaction mixture containing 1,3,6,8-tetrabromopyrene (1482 mg, 2.86 mmol, 1.0 eq.), 4-aminophenylboronic acid pinacol ester (3010 mg, 13.7 mmol, 4.8 eq.), K_2CO_3 (2175 mg, 15.7 mmol, 5.5 eq.), and $Pd(PPh_3)_4$ (330 mg, 0.29 mmol, 10 mol%) in 32 mL 1,4-dioxane and 8 mL degassed H_2O was refluxed at 115 °C for 3 d. After cooling to room temperature, H_2O (50 mL) was added. The resulting precipitate was collected via filtration and washed subsequently with H_2O (50 mL) and MeOH (100 mL). Recrystallization from 1,4-dioxane, followed by drying under high vacuum yielded the title compound, co-crystallized with approximately 1.5 dioxane molecules per formula unit, as a bright yellow powder (1792 mg, 2.56 mmol, 90%).

^{1}H NMR (400 MHz, DMSO-d_6): 8.13 (s, 4 H), 7.79 (s, 2 H), 7.34 (d, *J* = 8.4 Hz, 8 H), 6.77 (d, *J* = 8.5 Hz, 8 H), 5.30 (s, 8 H), 3.56 (s, 12 H, dioxane).

^{13}C NMR (100 MHz, DMSO-d_6): 148.2, 137.1, 131.0, 129.0, 127.6, 126.7, 126.1, 124.4, 113.9, 66.3 (dioxane).

1,3,6,8-tetrakis(4-formylphenyl)pyrene, Py(CHO)$_4$[10]

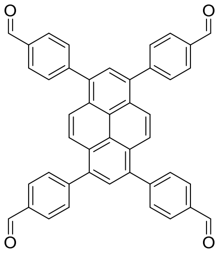

1,3,6,8-tetrabromopyrene (371 mg, 0.72 mmol, 1.0 eq.), 4-formylphenylboronic acid (480 mg, 3.20 mmol, 4.8 eq.), K_2CO_3 (547 mg, 3.66 mmol, 5.5 eq.) and $Pd(PPh_3)_4$ (83 mg, 0.066 mmol, 10 mol%) were refluxed at 115 °C in 8 mL 1,4-dioxane and 2 mL H_2O for 3 d. After cooling to room temperature, H_2O (20 mL) was added. The precipitate was collected by filtration and was washed with H_2O and MeOH. The resulting yellow powder was dried under vacuum (378 mg, 0.61 mmol, 85%).

^{1}H NMR (400 MHz, DMSO-d_6): 10.16 (s, 4 H), 8.18 (s, 4 H), 8.09 (d, *J* = 8.20 Hz, 8 H), 8.04 (s, 2 H), 7.86 (d, *J* = 8.20, 8 H).

Due to the low solubility of this compound in common deuterated solvents, no ^{13}C NMR spectra could be recorded.

C. COF syntheses

COF bulk powder syntheses were performed under argon atmosphere in PTFE-sealed glass reaction tubes (6 mL volume). Solvents and acetic acid were obtained in high-purity grades from commercial suppliers and were, unless shipped under argon, degassed and saturated with argon prior to use.

Py-TT COF. $Py(NH_2)_4$ (14.0 mg, 20 µmol, 1.0 eq.) and thieno-[3,2-*b*]thiophene-2,5-dicarboxaldehyde (7.8 mg, 40 µmol, 2.0 eq.) were filled into a reaction tube, followed by the addition of mesitylene (667 µL), benzyl alcohol (333 µL), and 6 M acetic acid (100 µL). The tube was sealed and kept at 120 °C for 3 d. After cooling to room temperature, the precipitate was collected by filtration, washed with MeCN and dried in air, yielding a bright red powder.

Py-1P COF. $Py(NH_2)_4$ (14.0 mg, 20 µmol, 1.0 eq.) and terephthalaldehyde (5.4 mg, 40 µmol, 2.0 eq.) were filled into a reaction tube, followed by the addition of mesitylene (667 µL), 1,4-dioxane (333 µL), and 6 M acetic acid (100 µL). The tube was sealed and heated at 120 °C for 3 d. After cooling to room temperature, the precipitate was collected by filtration, yielding a bright orange powder.

Py-Py COF. $Py(NH_2)_4$ (3.5 mg, 5.1 µmol, 1.02 eq.) and $Py(CHO)_4$ (3.1 mg, 5 µmol, 1.0 eq.) were filled into a reaction tube, followed by the addition of mesitylene (333 µL), benzyl alcohol (167 µL), and 6 M acetic acid (50 µL). The tube was sealed and kept at 120 °C for 3 d. After cooling to room temperature, the precipitate was collected by filtration, washed with MeCN and dried in air, yielding a bright yellow powder.

COF thin films were synthesized in 100 mL autoclaves equipped with a 28 mm diameter glass liner. Fused silica (Spectrosil 2000), sapphire (UQG Optics, *c*-axis cut), and ITO-coated glass (VisionTec, 12-15 ohms/sq) substrates were cleaned in detergent solution, water, acetone, and isopropanol, and activated with an O_2-plasma for 5 min directly before use. The substrates were placed horizontally in PTFE sample holders with the activated surface face-down.

Py-TT COF films. $Py(NH_2)_4$ (7.0 mg, 10 µmol, 1.0 eq.) and thieno-[3,2-*b*]thiophene-2,5-dicarboxaldehyde (4.0 mg, 20 µmol, 2.0 eq.) were filled into an autoclave, followed by the addition of mesitylene (1333 µL) and benzyl alcohol (666 µL). A substrate (fused silica, sapphire or ITO) was inserted, followed by the addition of 6 M acetic acid (200 µL). The autoclave was sealed and heated to 120 °C for 4 d. After cooling to room temperature, the substrate was immersed in dry MeCN and dried with compressed air. Thinner films were grown at shorter reaction times ranging from 4 h to 2 d.

Py-1P COF films. $Py(NH_2)_4$ (7.0 mg, 10 µmol, 1.0 eq.) and terephthalaldehyde (2.75 mg, 20 µmol, 2.0 eq.) were filled into an autoclave, followed by the addition of mesitylene (1333 µL) and benzyl alcohol (666 µL). An ITO substrate was inserted, followed by the addition of 6 M acetic acid (200 µL). The autoclave was sealed and heated to 120 °C for 3 d. After cooling to room temperature, the substrate was rinsed with dry MeCN and dried with compressed air.

Py-Py COF films. $Py(NH_2)_4$ (7.0 mg, 10 µmol, 1.0 eq.) and $Py(CHO)_4$ (6.2 mg, 10 µmol, 1.0 eq.) were filled into an autoclave, followed by the addition of mesitylene (1333 µL) and benzyl alcohol (666 µL). A substrate (sapphire or ITO) was inserted, followed by the addition of 6 M acetic acid (200 µL). The autoclave was sealed and heated to 120 °C for 3 d. After cooling to room temperature, the substrate was rinsed with dry MeCN and dried with compressed air.

Py-1P molecular fragment. The Py-1P molecular fragment was obtained by condensation of Py(NH2)4 (14 mg, 20 µmol, 1.0 eq.) with benzaldehyde (104 mg, 0.98 mmol, 49 eq.) in 2 mL CHCl3. After heating for 16 h at 70 °C, all volatiles were removed under high vacuum. The resulting precipitate was dissolved in DCM and crystallized by slow evaporation of the solvent.

The thin film of the molecular fragment was prepared by drop-casting a concentrated (~100 µM) DCM solution at room temperature, followed by drying in a static N2 atmosphere.

Amorphous Py-1P network. The amorphous Py-1P network was prepared by condensation of Py(NH2)4 (14 mg, 20 µmol, 1.0 eq.) with terephthalaldehyde (5.4 mg, 40 µmol, 2.0 eq.) in a solvent mixture containing mesitylene (900 µL), 1,4-dioxane (100 µL), and 6 M acetic acid (100 µL). The reaction mixture was heated at 70 °C for 5 min. After cooling to room temperature, the precipitate was collected by filtration, washed with 1,4-dioxane and DMF, and dried in air.

The thin film of the amorphous Py-1P network was grown under identical synthesis conditions as the Py-1P COF film, but employing a TiO2 (anatase) coated ITO substrate.[11]

Supplementary Figures

D. Py-1P COF structure analysis

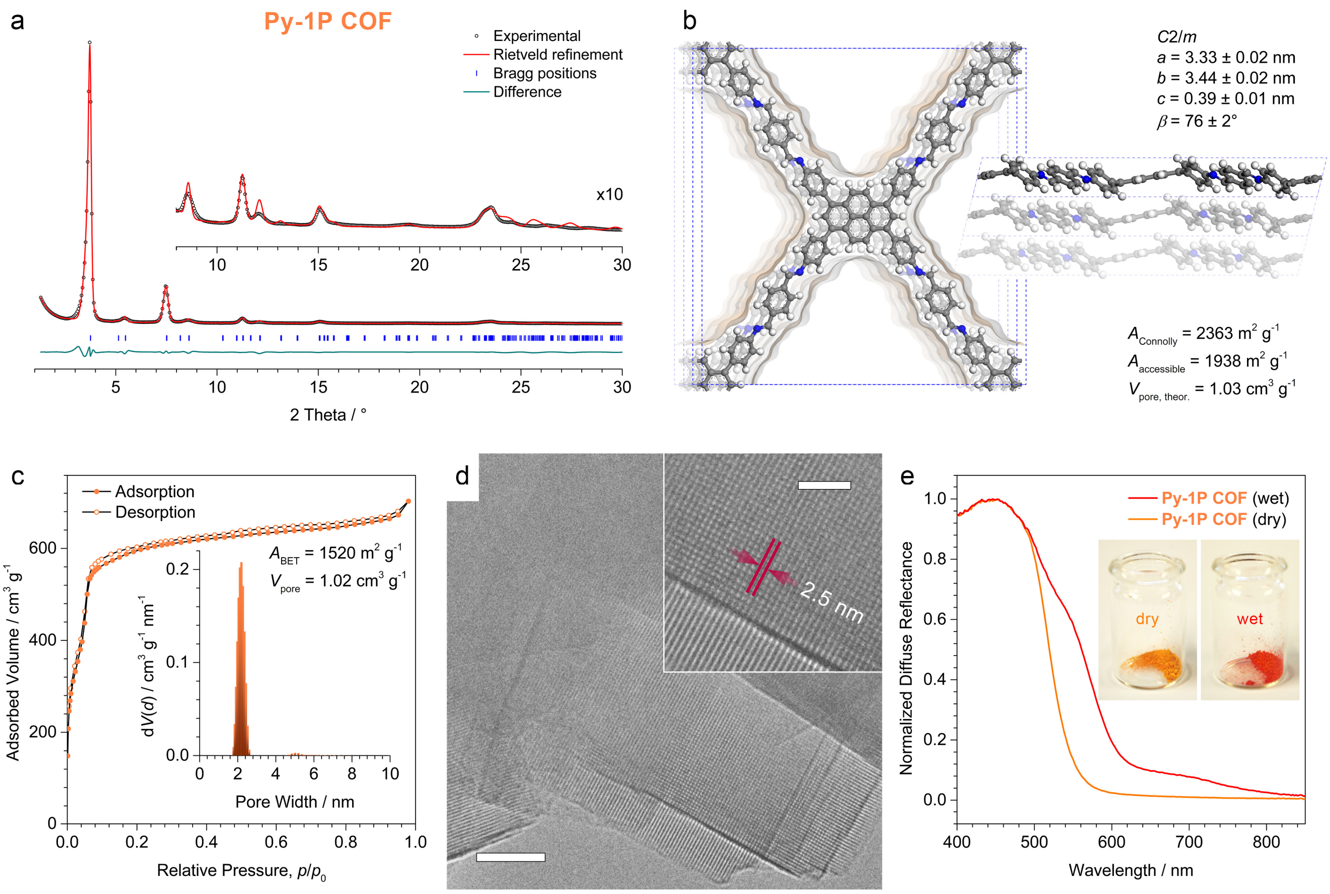

Supplementary Figure 1 | Structure analysis and solvatochromism of the Py-1P COF bulk powder. (**a**) Experimental PXRD pattern (black dots) of the Py-1P COF powder. Rietveld refinement (red line) using the structure model displayed in (**b**) provides a very good fit to the experimental data with only minimal differences between the experimental and the refined patterns (green line). R_{wp} = 3.4%, R_p = 8.4%. Bragg positions are indicated by blue ticks. Inset, magnified view of the 2θ > 8° region. (**b**) Top view (left) and side view (right) of the corresponding unit cell showing the pseudo-quadratic, offset-stacked structure that is typical for pyrene-based COFs. Crystallographic data are available as Supplementary Data 2. The structure has a Connolly surface of 2363 m^2 g^{-1}, an accessible surface area of 1938 m^2 g^{-1}, and a pore volume of 1.03 cm^3 g^{-1}. (**c**) Nitrogen sorption isotherm of the Py-1P COF recorded at 77 K. Inset, QSDFT calculation using an equilibrium model yields a very narrow pore-size distribution with a maximum at 2.1 nm. (**d**) High resolution TEM image showing the very large crystal domains of the Py-1P COF. Scale bar: 50 nm. Inset, magnified view onto a COF crystallite revealing the pseudo-quadratic arrangement of the COF pores with a periodicity of 2.5 ± 0.1 nm. Scale bar: 20 nm. (**e**) Diffuse reflectance spectra of the dry (orange) and water vapour saturated (red) Py-1P COF powder showing a strong solvatochromic red-shift.

The PXRD pattern of the Py-1P COF contains a number of sharp reflections including several well-defined higher-order reflections, indicating that this COF is equally well-crystallized as the Py-TT COF (Supplementary Figure 1a). Rietveld refinement in the monoclinic space group *C*2/*m* using the force-field optimized structure model shown in Supplementary Figure 1b provides a very good fit to the experimental data. As discussed in the main article, the large number of light atoms in the unit cell and the peak broadening due to the inherent flexibility of imine-linked COFs precludes the refinement of individual atom coordinates. We therefore observe minor deviations in the intensities of higher-index reflections that are primarily attributed to slight differences between the structure model and the actual COF structure.

N_2 sorption confirms that this COF is mesoporous with a pore-size distribution peaking at 2.1 nm (Supplementary Figure 1c). The total pore volume of 1.02 cm^3 g^{-1} derived from the sorption isotherm matches the theoretical porosity very well, indicating that the pores are open and accessible.

This COF forms particularly large crystallites that can reach several hundred nanometres in size and are terminated by well-defined facets (Supplementary Figure 1d). High-resolution TEM reveals the pseudo-quadratic geometry with a periodicity of 2.5 ± 0.1 nm, in excellent agreement with the pore-to-pore repeat distance of 2.4 nm in the refined structure model.

E. Py-Py COF structure analysis

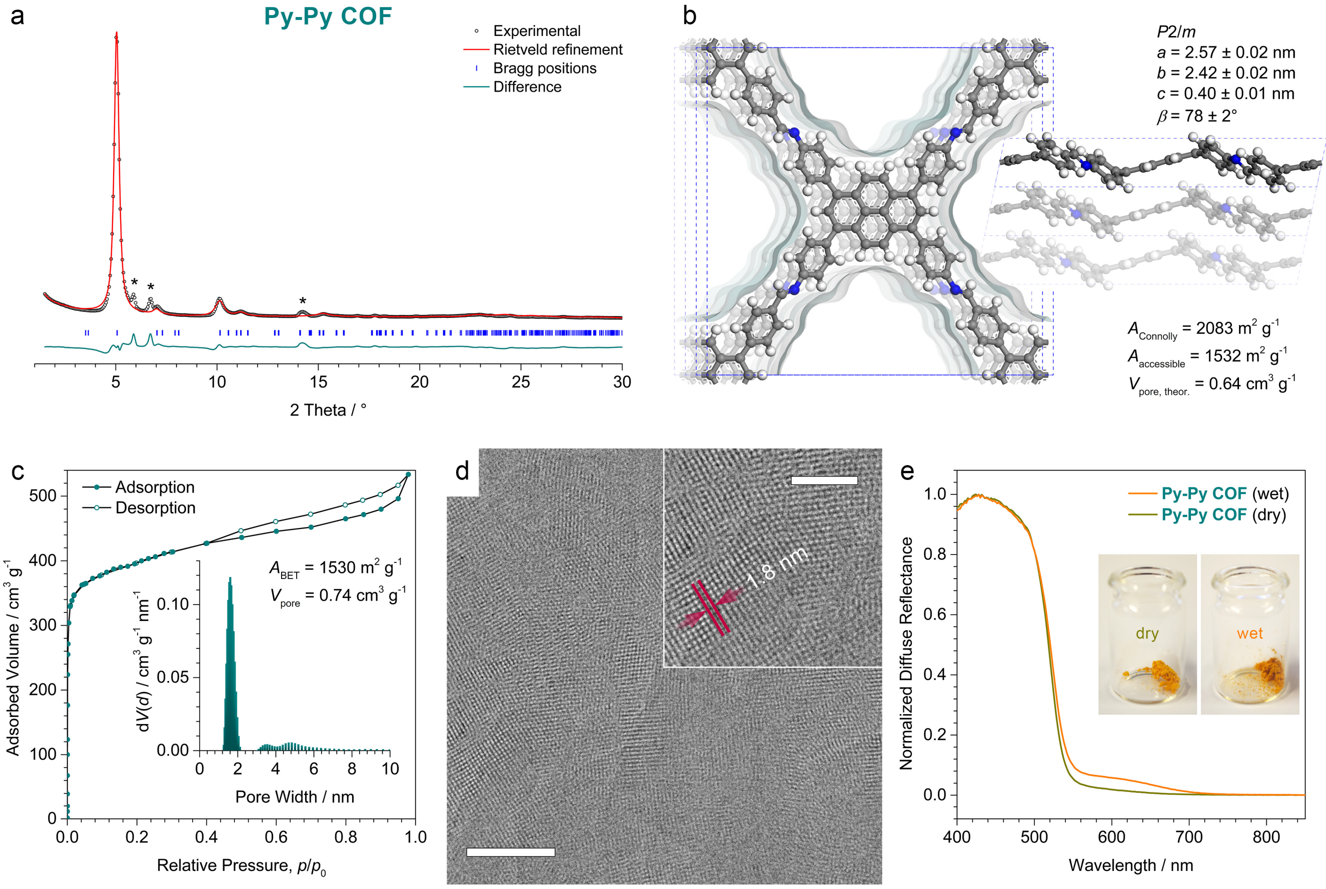

Supplementary Figure 2 | Structure analysis and solvatochromism of the Py-Py COF bulk powder. (**a**) Experimental PXRD pattern (black dots) of the Py-Py COF powder. The reflections marked with asterisks stem from residual Py(CHO)$_4$ starting material that could not be removed by washing due to its limited solubility. Rietveld refinement (red line) using the structure model displayed in (**b**) provides a good fit to the experimental data with only minor differences between the experimental and the refined patterns (green line). R_{wp} = 7.2%, R_p = 18.1%. Bragg positions are indicated by blue ticks. (**b**) Top view (left) and side view (right) of the corresponding unit cell showing the pseudo-quadratic, offset-stacked structure formed by alternating pyrene amine (centre) and aldehyde (corners) columns. Crystallographic data are available as Supplementary Data 3. The structure has a Connolly surface of 2083 m^2 g^{-1}, an accessible surface area of 1532 m^2 g^{-1}, and a pore volume of 0.64 cm^3 g^{-1}. (**c**) Nitrogen sorption isotherm of the Py-Py COF recorded at 77 K. Inset, QSDFT calculation using an equilibrium model yields a narrow pore-size distribution with a maximum at 1.6 nm. (**d**) High resolution TEM image showing the crystal domains of the Py-Py COF. Scale bar: 40 nm. Inset, magnified view onto a COF crystallite revealing the pseudo-quadratic arrangement of the COF pores with a periodicity of 1.8 ± 0.1 nm. Scale bar: 20 nm. (**e**) Diffuse reflectance spectra of the dry (green) and water vapour saturated (orange) Py-Py COF powder showing a small solvatochromic red-shift.

The Py-Py COF features a pseudo-quadratic overall geometry similar to the Py-TT and Py-1P COFs, but is composed of alternating columns of pyrene amine and pyrene aldehyde moieties. Rietveld refinement of the Py-Py COF in the space group *P*2/*m* yields a considerably smaller unit cell than for the other COFs in this study, in line with the reduced length of the pore walls due to the altered connectivity of this framework (Supplementary Figure 2a,b).

The consequently smaller pores give rise to a type I nitrogen sorption isotherm with a QSDFT pore-size distribution peaking at 1.6 nm (Supplementary Figure 2c).

High-resolution TEM confirms the anticipated pseudo-quadratic geometry of the framework with a periodicity of 1.8 ± 0.1 nm, in very good agreement with the pore-to-pore repeat distance of 1.7 nm in the refined structure model (Supplementary Figure 2d).

F. Py-TT COF film morphology

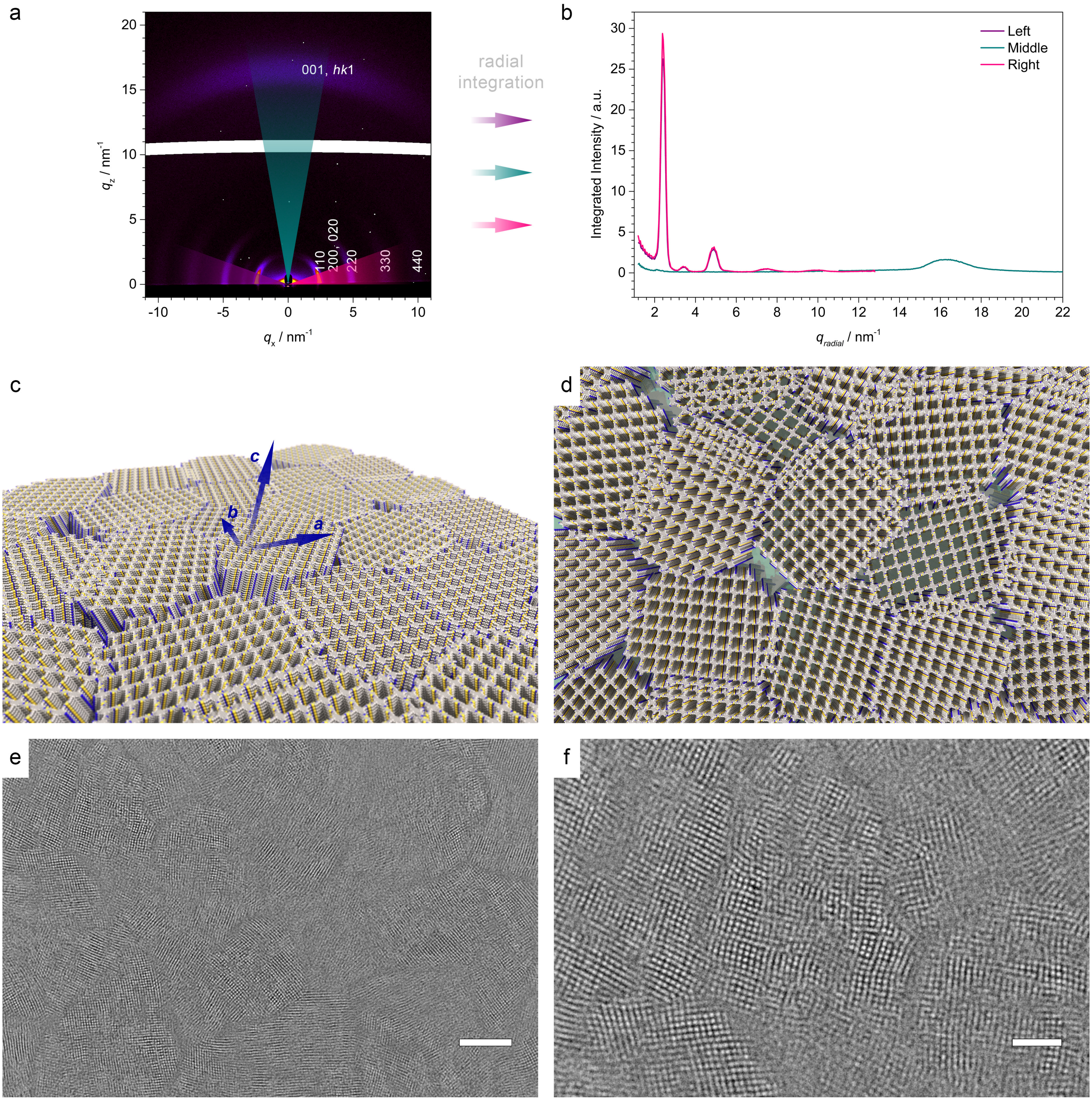

Supplementary Figure 3 | Morphology characterisation of the Py-TT COF films. (**a**) GIWAXS pattern of a 360 nm thick Py-TT COF film grown on a sapphire substrate. (**b**) Radial integration over the purple, green, and pink segments in the GIWAXS pattern (20° opening) shows that the COF film is highly textured with the crystallographic *ab*-plane parallel to the substrate. Most of the intensity of the 110, 020 and 200, 220, 330 and 440 reflections is confined to the segments directly above the sample horizon (purple and pink lines), while the middle segment contains the 001 reflection and contributions from other low-index *hk*1 reflections (green line; see discussion below). (**c**,**d**) Illustration of the polycrystalline film morphology. Individual COF domains grow with their *ab*-plane parallel to the substrate and at random rotation about the substrate normal. The unit cell axes are indicated for one crystallite. (**e**) High-resolution TEM image of Py-TT COF film revealing its polycrystalline, highly textured morphology with domain sizes around 50 nm. Scale bar: 50 nm. (**f**) Magnified view confirming the orientation of the pores towards the film surface. Scale bar: 20 nm.

The Py-TT COF films are highly textured with the imine-linked COF layers extending parallel to the substrate surface. This texture is identical for single-crystalline (*c*-cut sapphire), amorphous (fused silica), and polycrystalline (ITO) substrates, suggesting that the preferred orientation is generated by the anisotropy of the framework.[12,13] Consistently, individual COF domains grow at random rotation about the substrate normal (planar disorder) without any preferential alignment of their *a* and *b* axes with respect to the sapphire hexagonal plane. Due to the highly anisotropic unit cell, the *d*-spacings of the (001), (111) and other low-index (*hk*1) sets of lattice planes are almost identical. The corresponding reflections, however, appear under different angles relative to the substrate normal and give rise to an arc with $q \approx 16$ nm^{-1}.

G. Py-TT COF single carrier devices

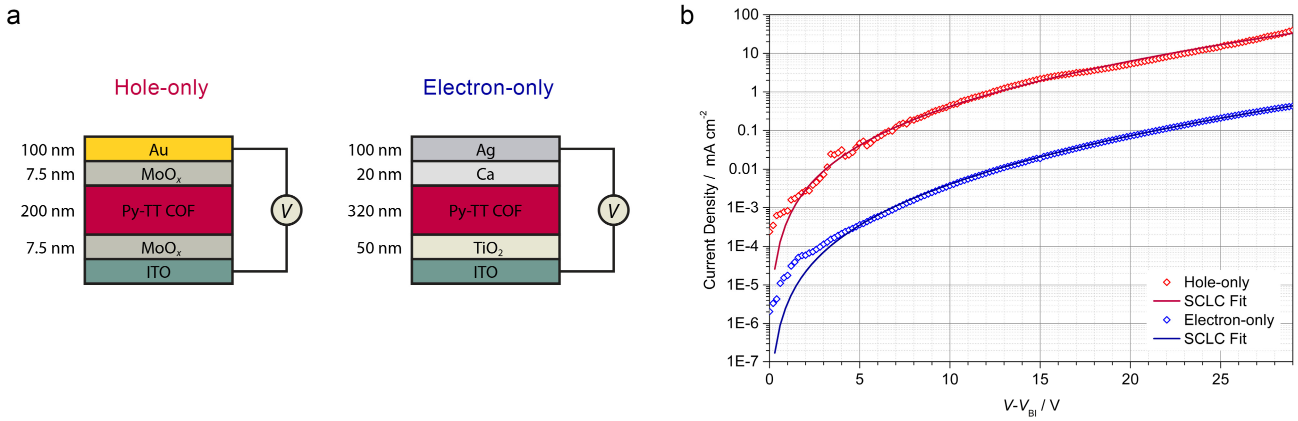

Supplementary Figure 4 | Electronic transport properties of Py-TT COF films. (**a**) Schematic illustrations of the hole-only and electron-only devices. (**b**) *J-V* curves of the single carrier devices measured in the dark (symbols) and the corresponding fits using the SCLC model (red and blue lines).

Owing to their sheet-like structure, the electronic properties of 2D COFs tend to be highly anisotropic with the highest conductivity and mobility typically along the π-stacked columns.[13-15] Electronic transport measurements can thus be used to draw conclusions about the COF crystallite orientation.

Vertical electronic transport was investigated by constructing single carrier devices. Py-TT COF films were grown as described above using MoO_x-coated (hole-only) or TiO_2-coated (electron-only) ITO substrates. The devices were completed by thermal evaporation of MoO_x and Au (hole-only), or Ca and Al (electron-only) electrodes through a shadow mask. COF layer thicknesses were determined from SEM cross-sections of the devices. The devices were measured in the dark with carrier injection from the top contact (MoO_x/Au or Ca/Ag, respectively).

Assuming Ohmic injection, the current in the devices will be space-charge-limited. In the space-charge-limited regime, the current density follows a modified Mott-Gurney law, taking into account the dependence of the mobility on the electric field (Poole-Frenkel model, exponential term in equation below).[16-18]

$$J(V) = \frac{9}{8}\varepsilon_0\varepsilon_r\mu_0\frac{(V - V_{BI})^2}{d^3}\exp\left(\beta\sqrt{\frac{(V - V_{BI})}{d}}\right)$$

ε_0	vacuum permittivity	μ_0	zero-field mobility	d	COF layer thickness
ε_r	relative permittivity	V_{BI}	built-in voltage	β	field effect mobility coefficient

Supplementary Table 1 | SCLC analysis of the Py-TT COF single-carrier devices.

	μ_0 / $cm^2 V^{-1} s^{-1}$	ε_r (fixed)	V_{BI} / V*	d / nm	β / $cm^{0.5} V^{-0.5}$	Adjusted R^2
hole-only	$(4.02 \pm 0.04) \times 10^{-6}$	3.5	1.4	200	4.5×10^{-3}	0.984
electron-only	$(1.02 \pm 0.01) \times 10^{-7}$	3.5	4.2	320	6.5×10^{-3}	0.999

*The built-in voltages were extrapolated from the *J-V* curves. The built-in voltage of the hole-only device could stem from a slight modification of the oxygen stoichiometry (de-doping) of the bottom MoO_x layer during the solvothermal COF synthesis.

To the best of our knowledge, this represents the highest hole-only SCLC mobility of any COF to date,[15] and also is the first measurement of a vertical transport electron mobility.

Lateral transport measurements across gap electrodes deposited on either on the COF film surface (200 µm gap) or on the substrate (50 µm gap) did not show any measurable conductivity, suggesting that the highest conductivity is indeed along the *π*-stacked columns and that the orientation of the COF crystallites is unchanged throughout the entire film.

H. Solvent-induced spectral changes

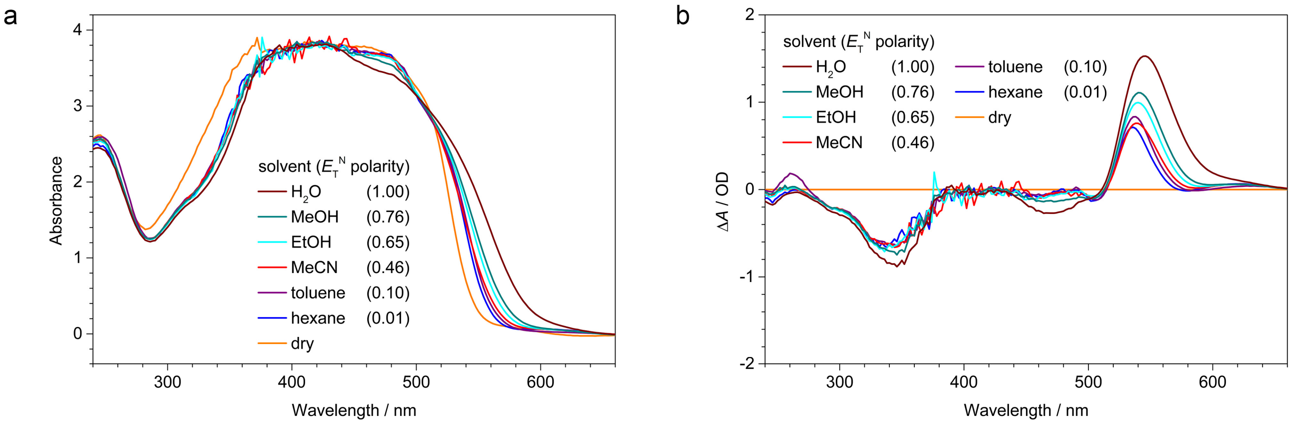

Supplementary Figure 5 | Solvent-induced absorption changes of the Py-TT COF. (**a**) UV-Vis spectra of the Py-TT COF film exposed to different solvent-saturated N_2 streams. (**b**) Plot of the absorption change, $A_{solvent}$ - A_{dry}, showing a strong solvent-induced absorbance in the 520-640 nm region that is accompanied by two bleach bands extending over the 440-500 and 280-380 nm regions. The magnitude of the solvent-induced spectral changes increases with increasing E_T^N polarity of the respective solvent.

I. Thickness-dependent response times

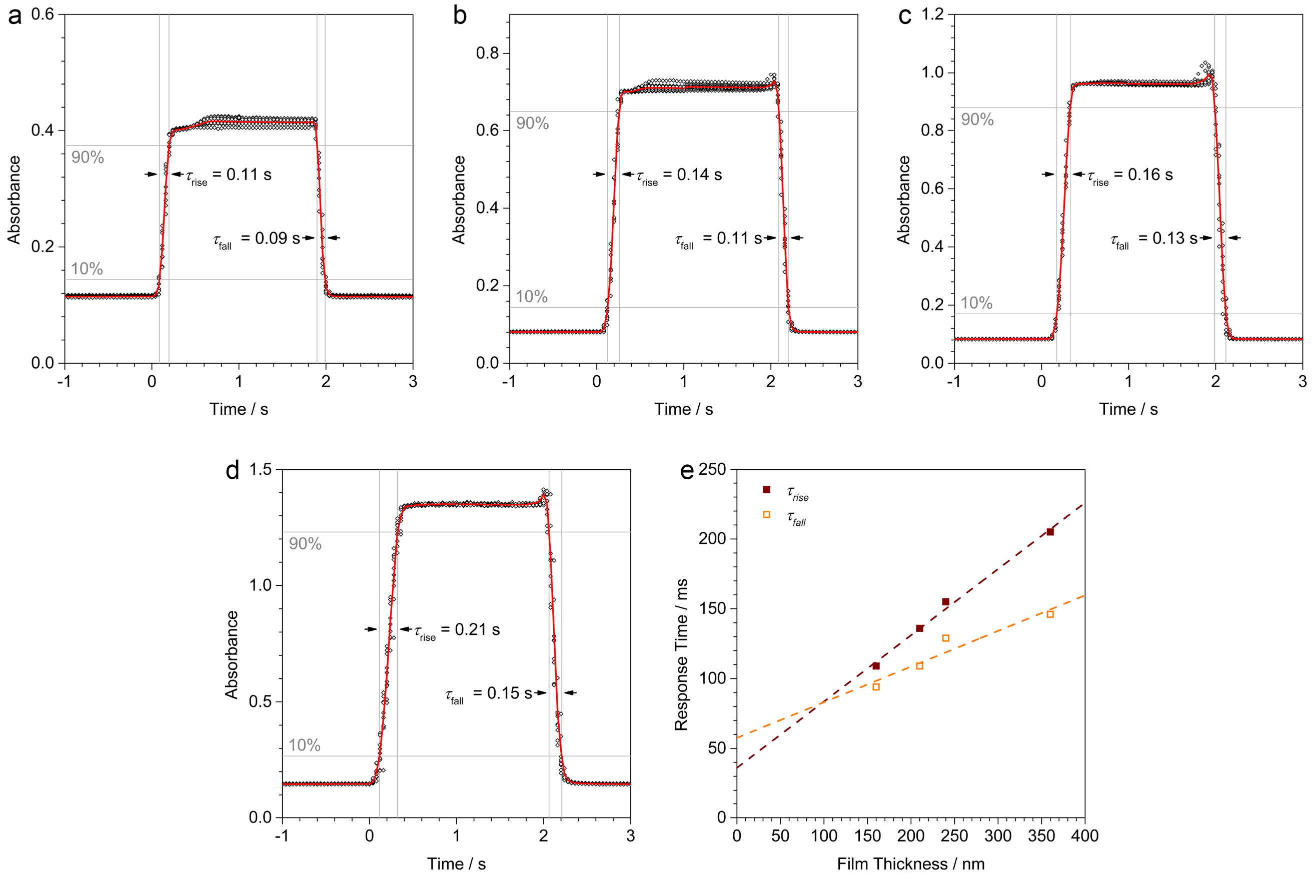

Supplementary Figure 6 | Film thickness-dependent response times of oriented Py-TT COF films. (**a-d**) Response times at λ = 560 nm derived from the averages (red lines) of 10 humidity cycles (dry to water-saturated). COF film thicknesses of 160, 210, 240, and 360 nm were realized via reaction times of 4 h, 1 d, 2 d, and 4 d, respectively. The spikes at the end of each “wet” period stem from pressure fluctuations during switching of the gas streams that cause a momentary increase of the water content in the COF film. (**e**) Correlation between the COF film thickness and the respective response times. The data might contain an offset of 30 - 50 ms that is associated with experimental factors, such as a temporary mixing of the dry and water-saturated gas flows during switching. The dashed lines are added as a guide to the eye.

J. Reproducibility and stability

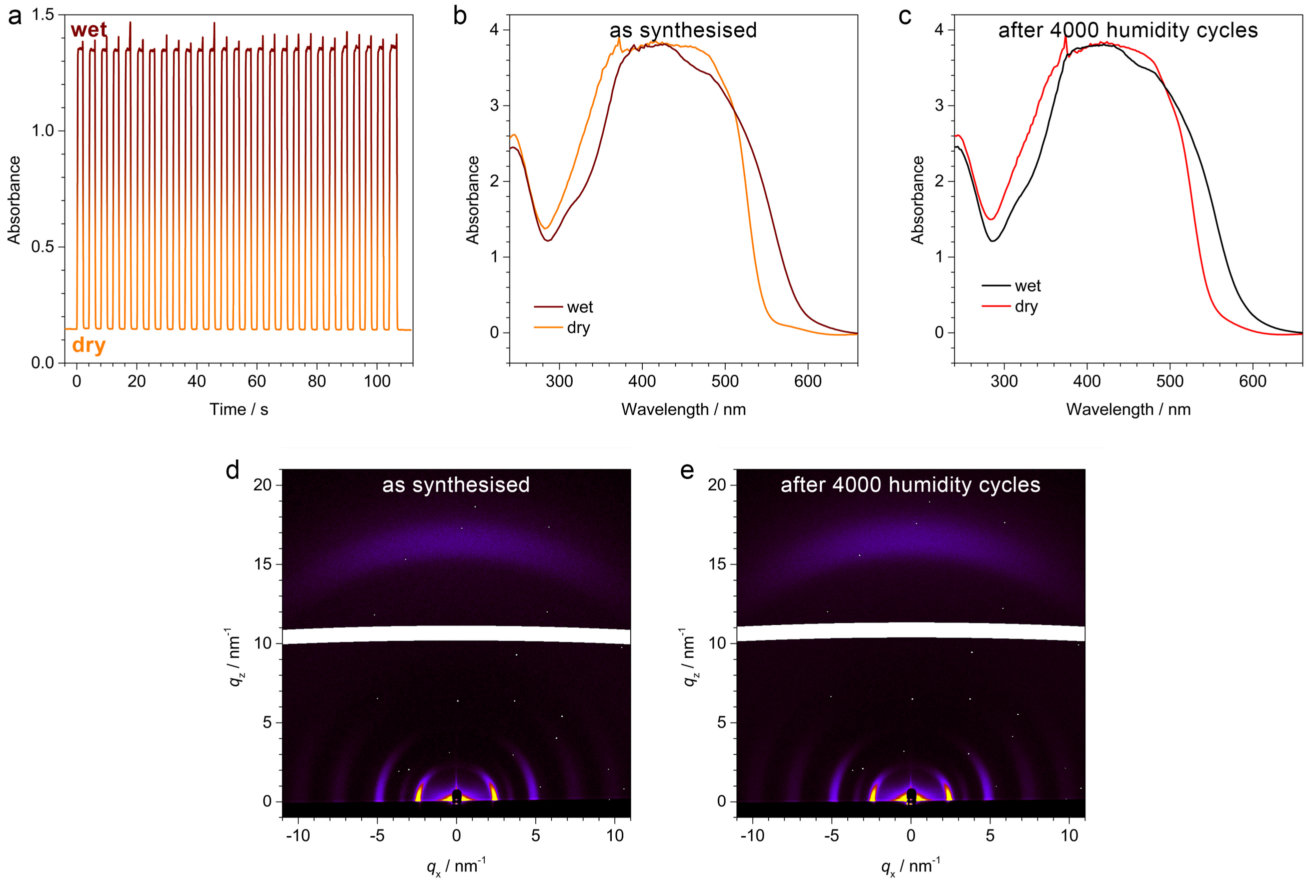

Supplementary Figure 7 | Reproducibility of the solvatochromic response and stability of the Py-TT COF films towards repeated humidity cycling and storage in ambient air. (**a**) Repeated humidity cycling (dry to water-saturated) underlines the complete reversibility of the solvatochromic response of the COF film. The spikes at the end of each "wet" period stem from pressure fluctuations during switching of the gas streams that cause a momentary increase of the water content in the COF film. (**b**,**c**) Absorption spectra of the as-synthesized Py-TT COF film and the same sample after 4000 humidity cycles and storage in ambient air for 250 d. The optical properties and solvatochromic response remain virtually unchanged. (**d**,**e**) The corresponding GIWAXS patterns confirm that the crystallinity and texture are fully retained. The white dots originate from damaged and deactivated pixels on the detector.

K. Py-TT COF photoluminescence spectra

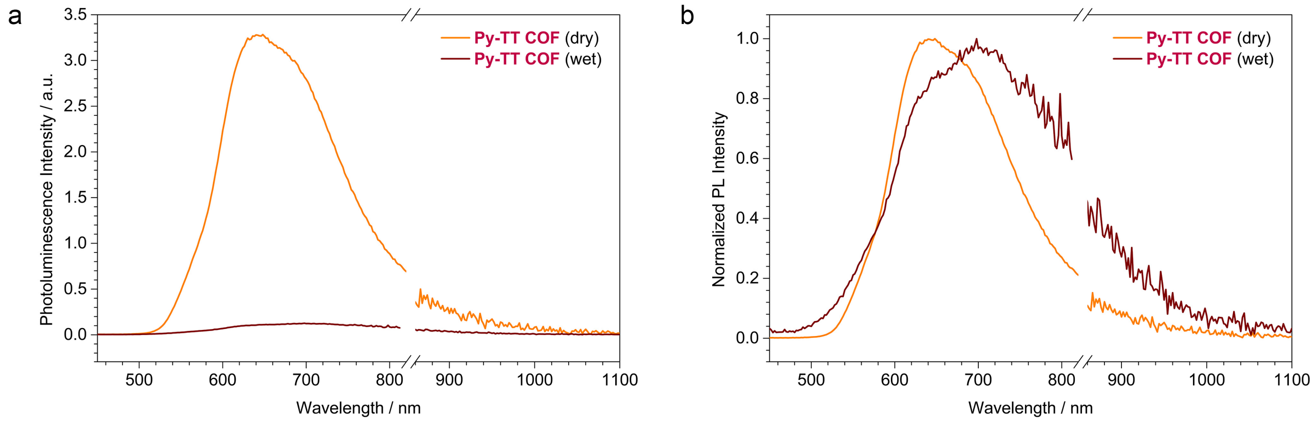

Supplementary Figure 8 | PL spectra of the dry and water-saturated Py-TT COF. (**a**) The dry Py-TT COF exhibits a moderately intense red PL, which is quenched by more than 95% upon exposure to humid atmosphere. (**b**) The normalized representation of the spectra reveals that this quenching is accompanied by a spectral shift towards lower energies, indicating changes in the electronic structure of the emissive states.

L. Humidity-dependent PXRD analysis

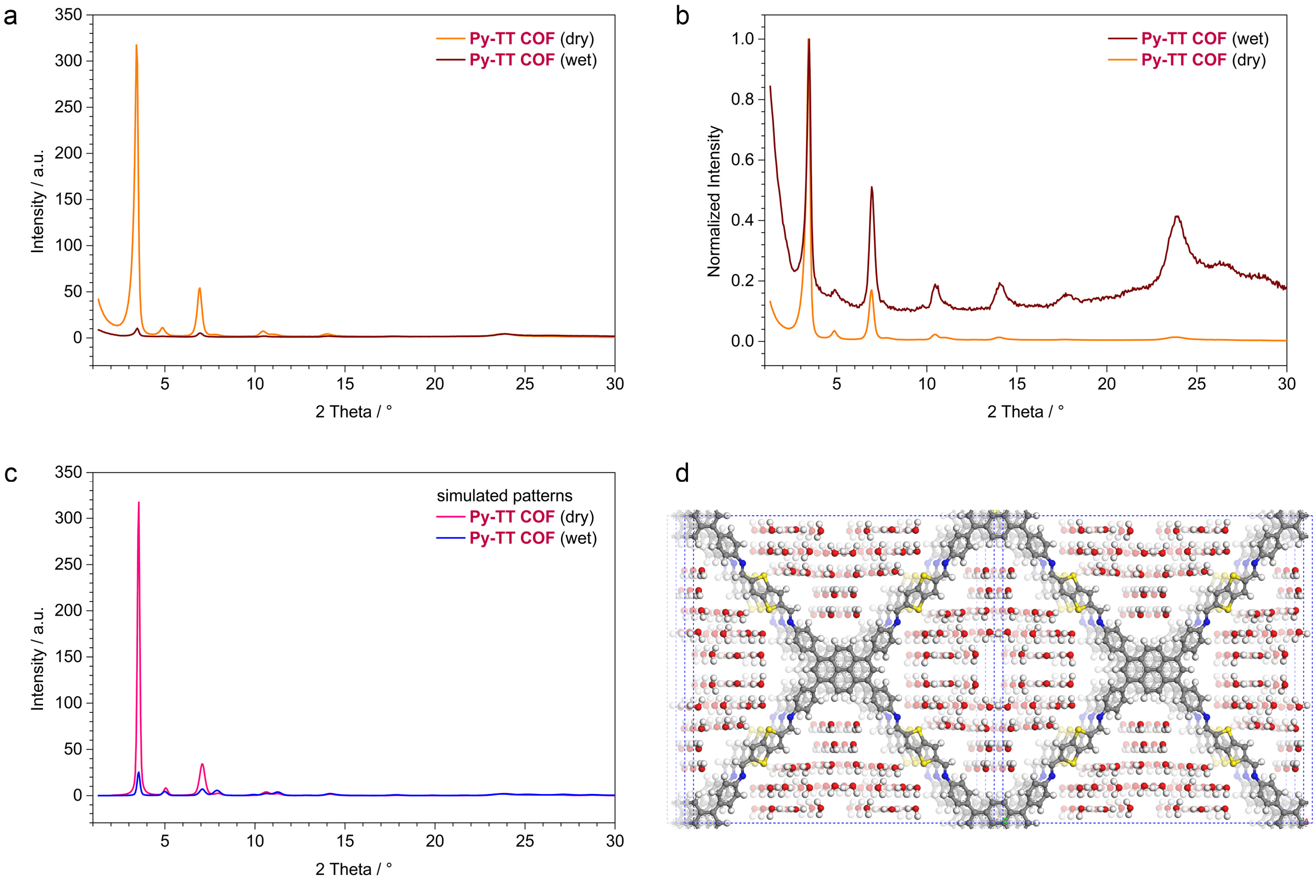

Supplementary Figure 9 | Comparison of the Py-TT COF PXRD patterns recorded in the dry and water-saturated states. (**a**) PXRD patterns of the dry (orange) and the water vapour-saturated Py-TT COF (brown). The reflection intensities, in particular at low angles, are significantly reduced in the water-saturated material. (**b**) The normalized representation of the PXRD patterns indicates no structural changes upon exposure to humidity as the reflection positions remain identical. (**c**,**d**) The observed reduction of reflection intensities can be reproduced in silico by filling the pores of the Py-TT COF structure model with water molecules.

M. Solid-state NMR spectroscopy

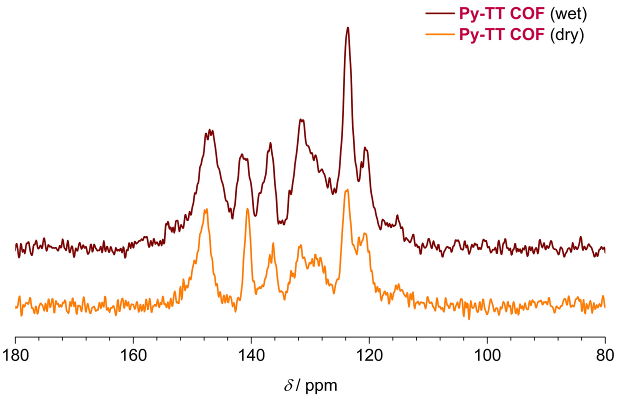

Supplementary Figure 10 | $^{13}C(^{1}H)$ cross-polarisation (cp) magic angle spinning (MAS) solid-state NMR spectra of Py-TT COF powder in the dry and water-saturated states. The spectrum exhibits a series of signals in the 110-160 ppm range originating from the 13 aromatic carbon atoms in the framework. Interactions with water molecules in the water-saturated framework might be the cause of minor changes in some peak intensities, but no shifts that would indicate an altered chemical environment are observed.

N. Analysis of the Py-1P and Py-Py COF films

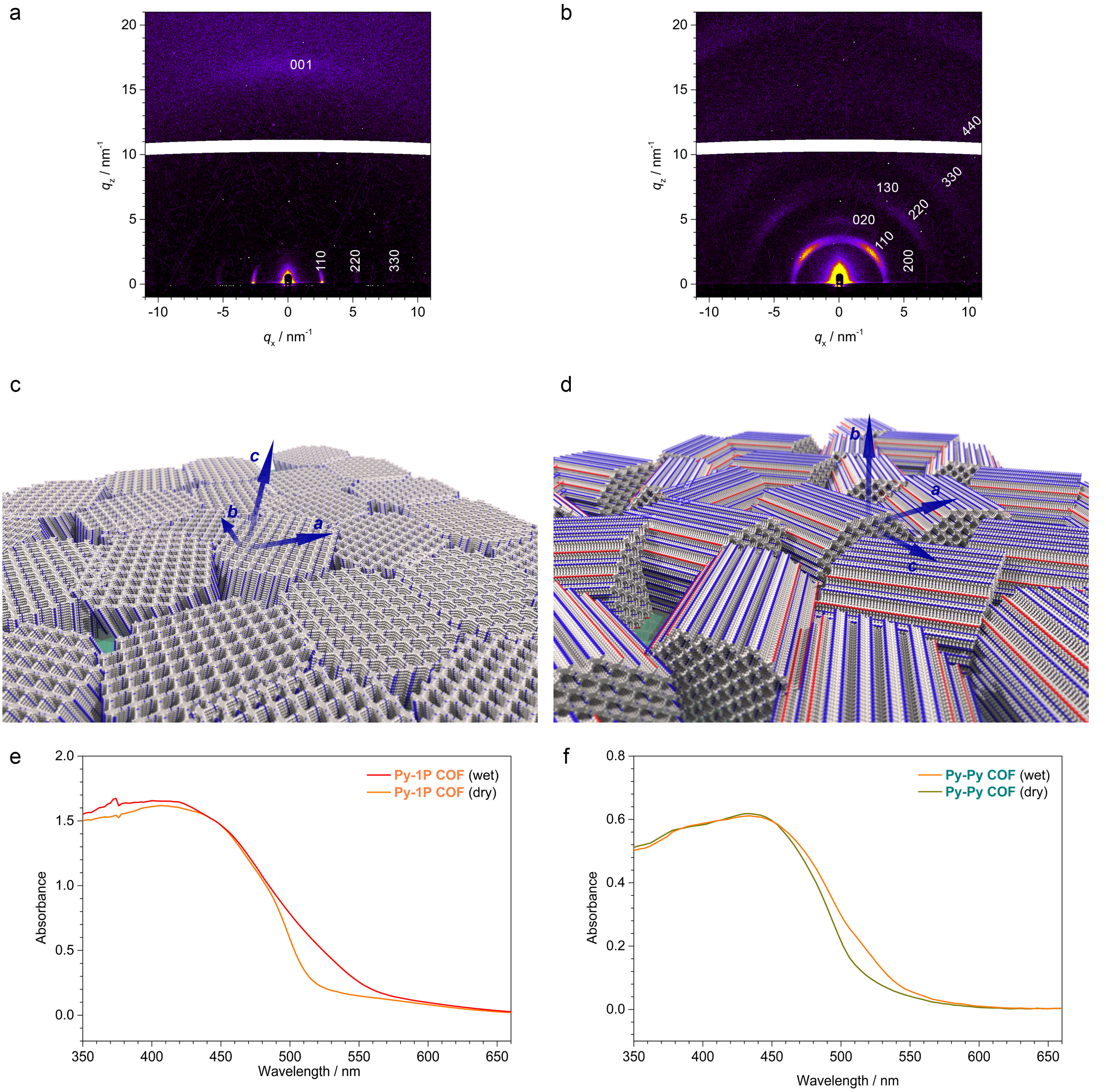

Supplementary Figure 11 | Structure characterisation and solvatochromism of Py-1P and Py-Py COF films. (**a**) GIWAXS pattern of a Py-1P COF film grown on an ITO substrate. The intensity of *hk*0 reflections is concentrated directly above the sample horizon, whereas the 001 reflection appears close to the substrate normal. This indicates that the Py-1P COF film grows with the COF layers parallel to the substrate. (**b**) GIWAXS pattern of a Py-Py COF film grown on a sapphire substrate. In contrast to the other COFs in this study, this framework grows with the *ac*-plane parallel to the substrate, possibly due to interactions of the aldehyde building block with the substrate. (**c**,**d**) Illustrations of the polycrystalline Py-1P and Py-Py COF films. The unit cell axes are indicated for one crystallite. (**e**,**f**) Solvatochromic response of the COF films towards a water-saturated atmosphere. Both COFs exhibit a humidity-induced absorption in the 480-560 nm region, which is, however, much narrower and less pronounced than observed for the Py-TT COF.

O. Protonation experiments

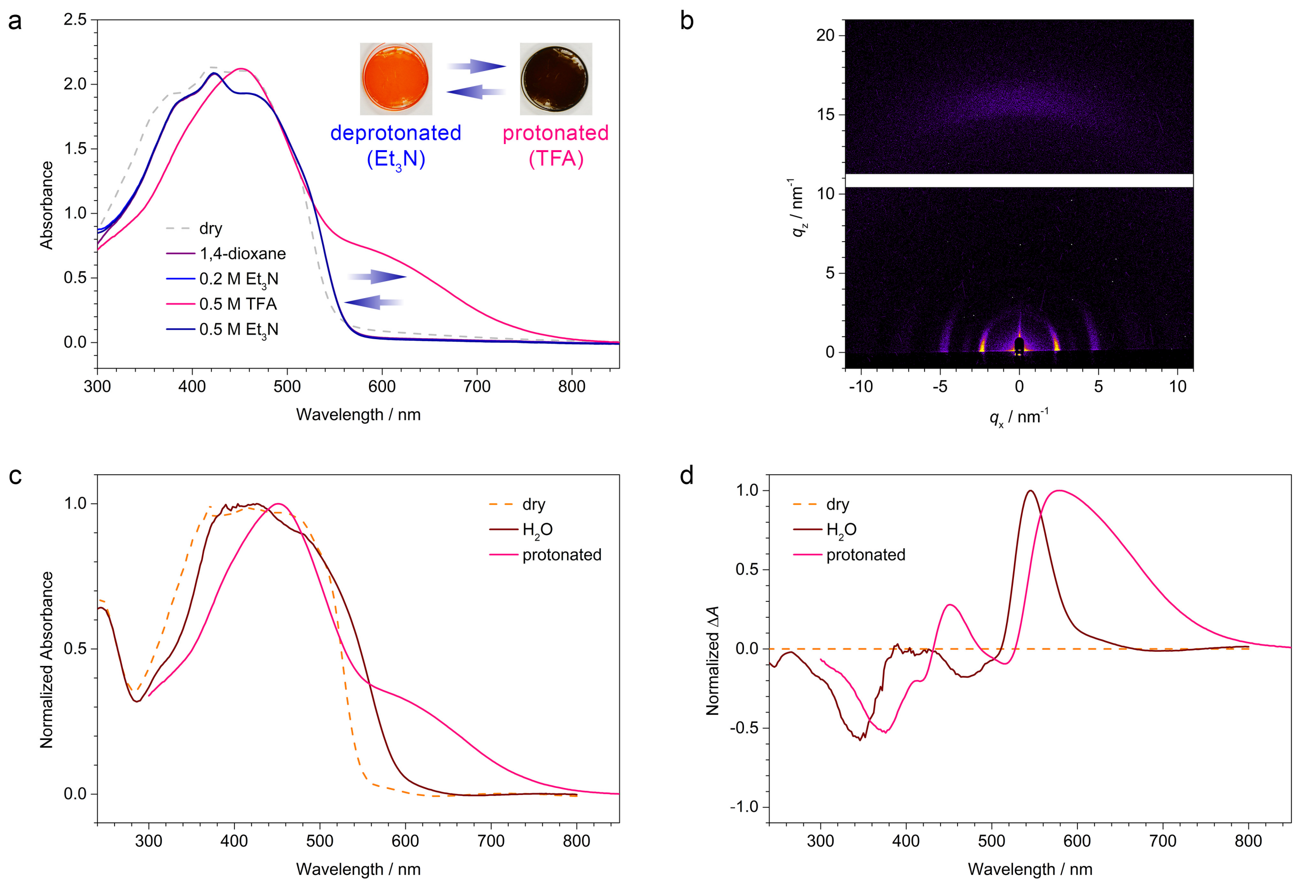

Supplementary Figure 12 | Reversible protonation and deprotonation of a Py-TT COF film. (**a**) The transmission UV-Vis spectra of a dry COF film (grey) and the same film immersed in 1,4-dioxane (purple) and a Et_3N/1,4-dioxane solution (light blue) confirm that the imine bonds of the as-synthesized COF are not protonated. However, they can be reversibly protonated by immersion in TFA solution (pink), and subsequently deprotonated with Et_3N solution (dark blue). (**b**) GISAXS pattern of the Py-TT COF film after five protonation-deprotonation cycles. (**c**) Comparison of the absorption spectra of a dry Py-TT COF film (orange), the same sample in water-saturated atmosphere (brown), and the protonated Py-TT COF (pink). (**d**) The corresponding humidity- and protonation-induced spectral changes. Since the protonation and the humidity experiments were carried out with two different COF films (200 nm and 360 nm thick, respectively), the data presented in subfigures **c** and **d** are normalized.

The above protonation/deprotonation experiment confirms that the as-synthesized COFs reported in this study are non-protonated, despite their acid-catalysed polymerisation (Supplementary Figure 12a). Protonation of the imine bonds, however, can be achieved with trifluoroacetic acid (TFA). The resulting immediate colour change from orange to dark brown stems from an additional absorption band that extends to about 800 nm as well as a modified absorption in the 300-550 nm range. The crystalline framework remains intact during protonation/deprotonation (Supplementary Figure 12b).

Although hydrogen bonding between the dye and solvent molecules is considered to be one of the contributions to the solvatochromic effect in protic solvents, and is likely to happen also with our COFs, the actual protonation of the imine bond through reaction with a strong acid produces a qualitatively different and much more red-shifted spectrum (Supplementary Figure 12c,d).

P. Py-1P molecular fragments and amorphous networks

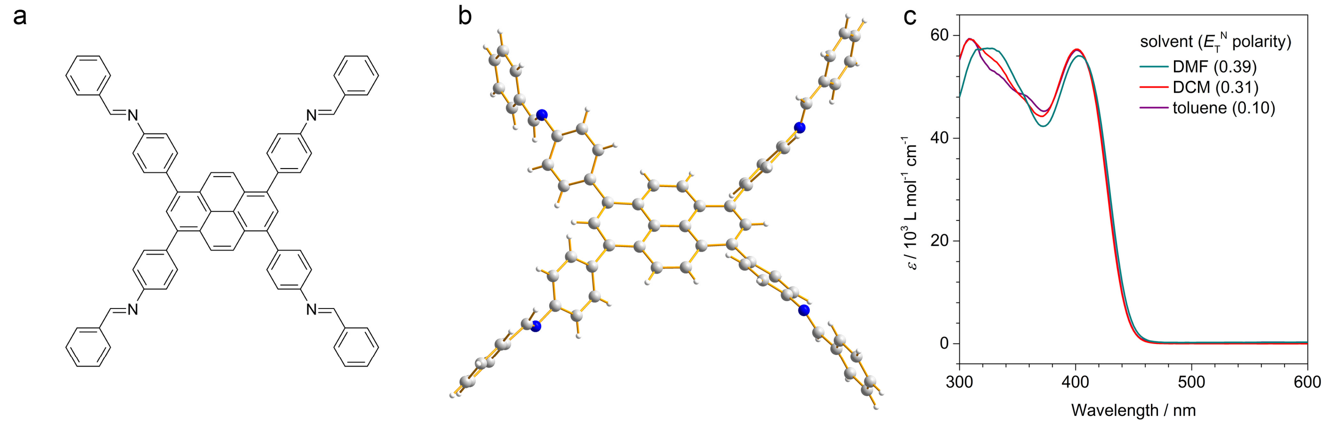

Supplementary Figure 13 | Solvatochromic response of a Py-1P molecular fragment. (**a**) Chemical structure of the Py-1P molecular fragment. (**b**) The corresponding single crystal structure. X-ray structure data are taken from ref 8. (**c**) Solvatochromism of the Py-1P fragment in solution. Concentration: 20 µM for DMF and toluene, 25 µM for DCM.

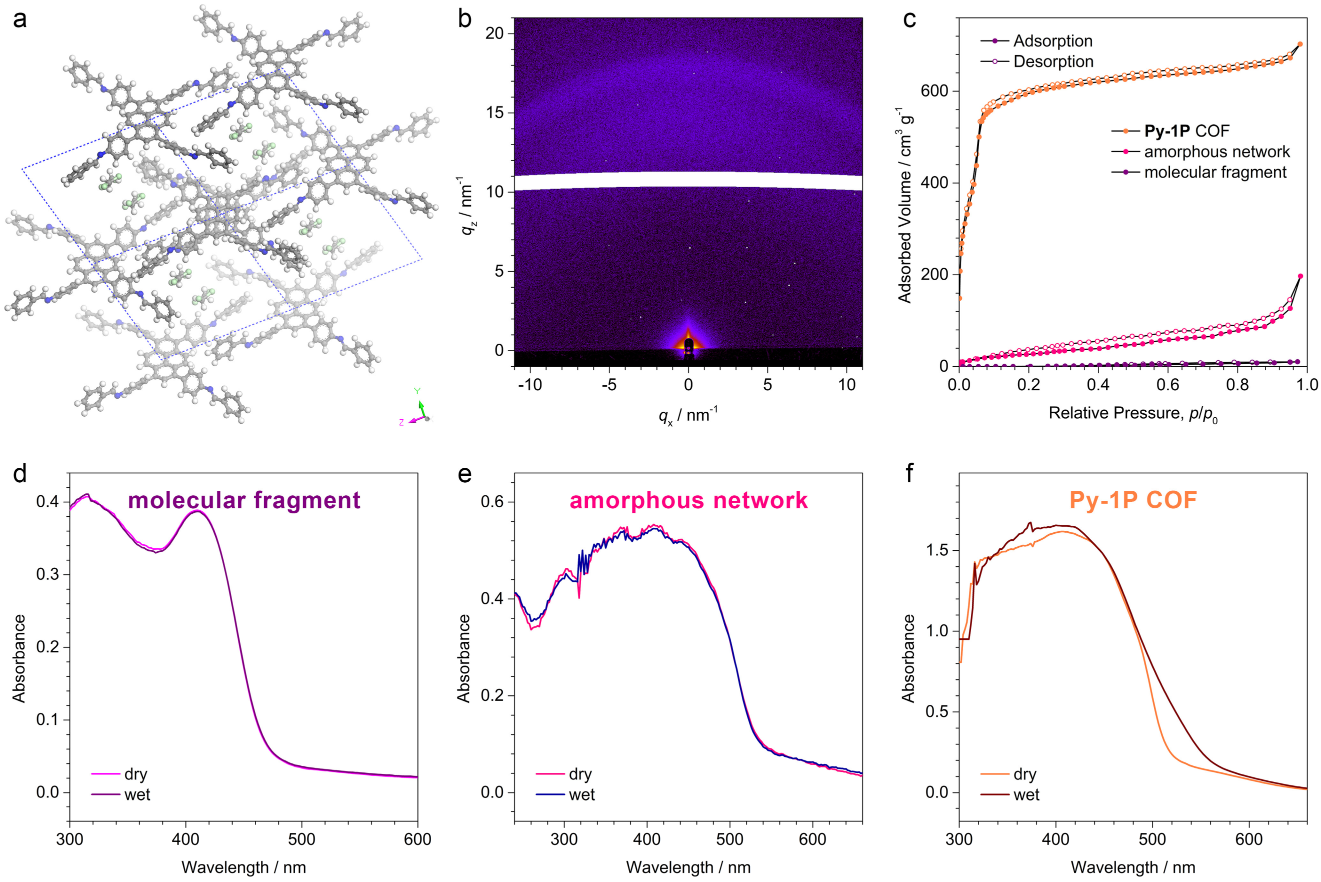

Supplementary Figure 14 | Porosity and solvatochromic response of Py-1P-type materials. (**a**) The Py-1P molecular fragment crystallizes in slip-stacked columns with two co-crystallized DCM molecules per formula unit, rendering the material completely non-porous. (**b**) GISAXS pattern of an amorphous Py-1P network thin film grown on TiO_2-coated ITO. The diffuse arcs at q = 14 and q = 17.5 originate from the substrate. (**c**) N_2 sorption isotherms of the three Py-1P-type compounds. While the amorphous network displays some N_2 uptake at higher relative pressures (corresponding to large pores and textural porosity), the crystallized molecular fragment is non-porous. (**d**-**f**) Solvatochromic response of the three Py-1P-type thin films when exposed to a water-saturated atmosphere. Despite them being chemically almost identical, only the crystalline COF is accessible for the water molecules and hence displays a measurable colour change.

In order to investigate the influence of the electronic structure and accessibility in thin films, we chose to compare the solvatochromism of the COF with an amorphous network derived from the same building blocks and a molecular model compound. We selected the Py-1P family of compounds for this study, since in this case all three modifications are synthetically accessible (see below).

The molecular fragment consisting of a tetraphenylpyrene centre with four imine-linked phenyls (Supplementary Figure 13a,b) displays a weak solvatochromism in the 300-400 nm range when dissolved in organic solvents (Supplementary Figure 13c). This is expected considering the moderate charge-transfer character of optical transitions from the electron rich core to the accepting imines. When deposited as a solid film, however, this material does not exhibit any measurable solvatochromic effect towards water vapour (Supplementary Figure 14d). We attribute this to the completely non-porous solid-state structure (Supplementary Figure 14a) and hence strongly restricted access of the water atmosphere to the bulk of the material. The co-crystallized DCM is bound strongly enough such that it is not removed during the degassing (120 °C, 24 h, turbo-pumped vacuum) prior to the sorption measurements (Supplementary Figure 14c).

The importance of molecular access for a measurable solvatochromism is further highlighted by comparing the Py-1P COF with an amorphous Py-1P network (Supplementary Figure 14b). Despite being chemically identical to the crystalline COF, the latter does not display any observable solvatochromic colour change (Supplementary Figure 14e). While the amorphous network shows some degree of porosity in the nitrogen sorption experiment, this is only observed at higher partial pressures corresponding to large voids or textural porosity. In the mesoporous region, which governs the access of guest species on a molecular level, the porosity is almost zero.

In summary, while their chemical structure defines to what extent the materials can be solvatochromic, our above experiments show that it is the accessibility on a molecular length scale that governs whether this solvatochromism is actually displayed by the respective solid-state material. Only the crystalline COFs with their well-ordered mesoporosity are able to deliver a strong and fast solvatochromic response.

Q. Additional COF characterisation

Thermogravimetric analysis

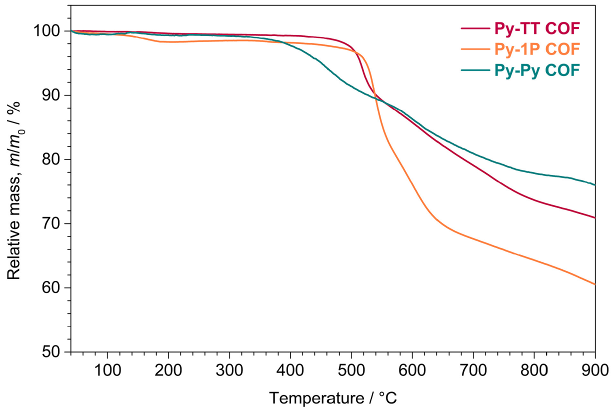

Supplementary Figure 15 | Thermogravimetric analysis. Thermogravimetric analysis of the three COFs measured under an N_2 atmosphere at a heating rate of 1 K min^{-1}. Thermal decomposition starts only at 350 °C in the Py-Py COF. The Py-TT and Py-1P COFs display an even higher stability with decomposition temperatures above 500 °C, placing them among the most stable COFs to date.[19,20]

IR spectroscopy

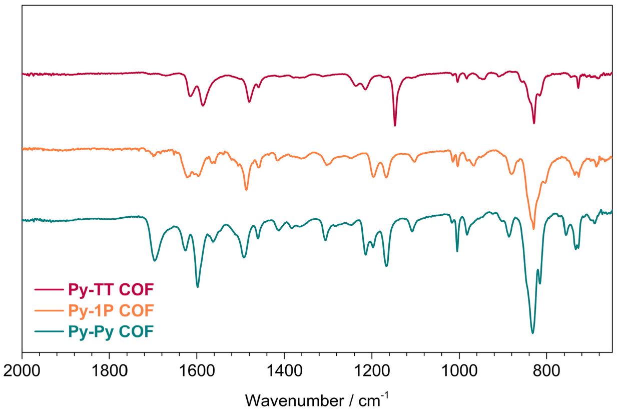

Supplementary Figure 16 | Fourier-transform IR spectra of the COF powders. IR spectroscopy confirms the formation of imine-linked frameworks with characteristic imine vibration bands around 1620 cm^{-1}.

Supplementary Table 2 | Imine C=N stretching vibration frequencies.

	ν(C=N) / cm^{-1}
Py-TT COF	1615
Py-1P COF	1621
Py-Py COF	1626

R. Py-TT COF based humidity sensor

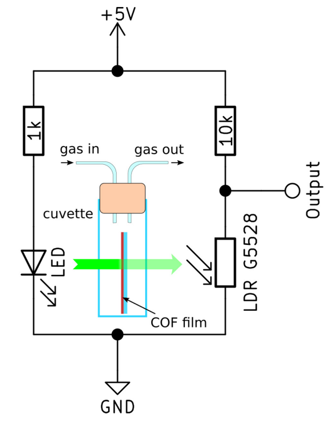

Supplementary Figure 17 | Circuit diagram of the COF-based humidity sensor. The light emitted from the green LED is transmitted through the Py-TT COF film and detected via a light-dependent resistor. In a dry atmosphere, the COF is almost transparent to green light, whereas it becomes increasingly opaque with increasing humidity.

To demonstrate the applicability of a solvatochromic COF film for sensing, we constructed a simple humidity sensor based on the circuit shown in Supplementary Figure 15. The Py-TT COF film acts as a humidity-responsive attenuator to the light emitted from a common 5 mm green InGaN LED. Detection of the transmitted light is achieved by a CdS-based light-dependent resistor (LDR, type: GL5528, peak response at 540 nm) connected as a variable voltage divider with a 10 kΩ resistor. Both LED and LDR are shielded with black heat shrink tubing to minimize ambient light effects. The COF film is placed between the LED and LDR in an optical cuvette equipped with a gas inlet and outlet.

The analog output signal can be probed between the LDR and the 10 kΩ resistor. We used a development board equipped with an ATMEGA328P microcontroller for read-out and digitalisation. This signal can be monitored on a PC in real-time or be logged over an extended period of time. The microcontroller was programmed using the Arduino IDE (v. 1.6.5) and the graphical display was programmed in Processing (v. 3.3.5).

A video of the Py-TT COF humidity sensor in operation is available as Supplementary Movie.

S. TD-DFT simulations

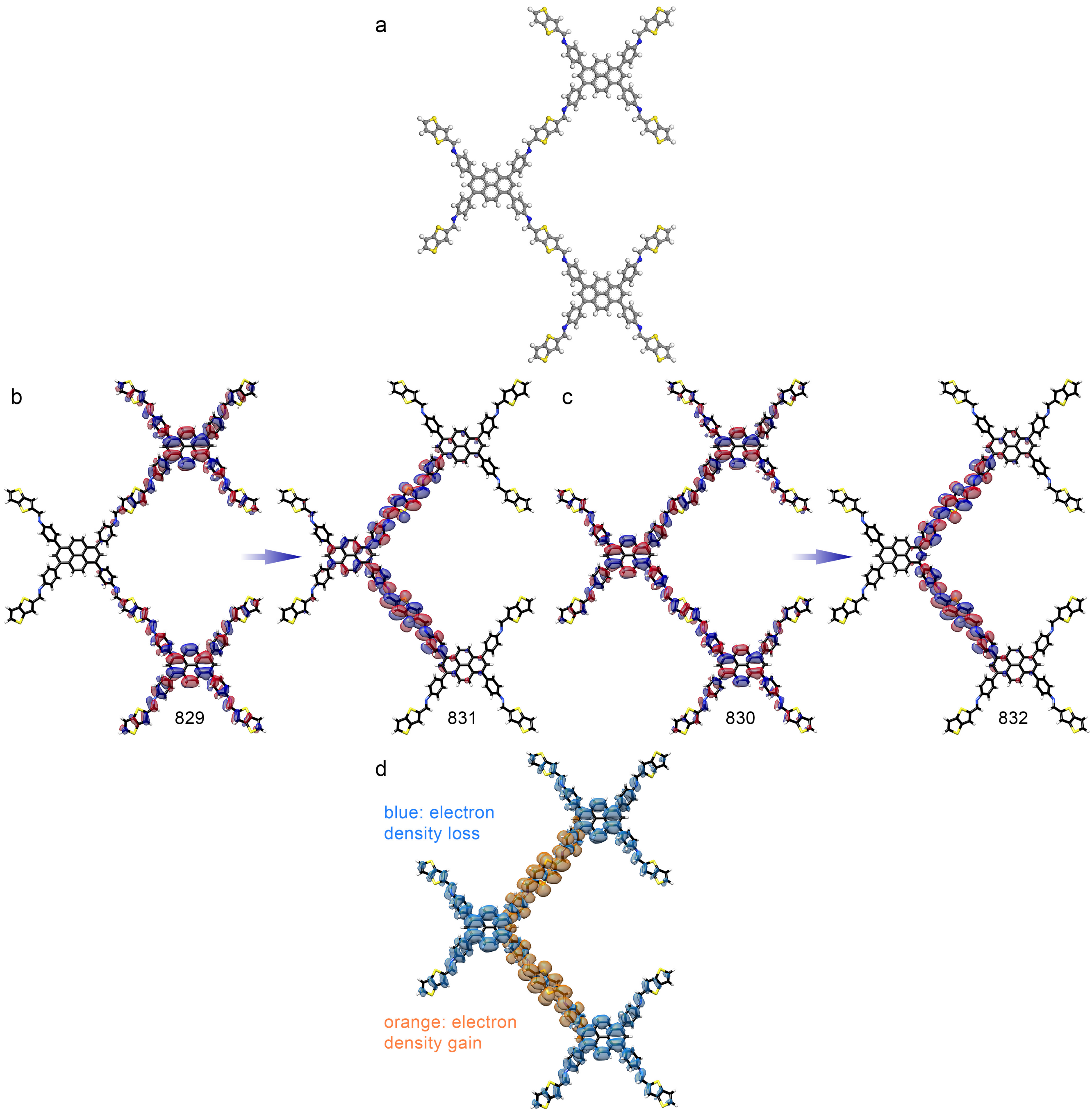

Supplementary Figure 18 | TD-DFT-simulation of the first excited state. (**a**) The single-layer Py-TT COF fragment used in the TD-DFT calculations. (**b**,**c**) In vacuum, the first excited state can be described by the one-electron transitions between two pseudo-degenerate pairs of orbitals. Orbital isosurfaces are drawn at an isovalue of 0.01. (**d**) The electron density difference plot reveals that the transition from the ground state to the first excited state involves a charge-transfer from the pyrenes to the thienothiophene bridges (orange = electron density gain, blue = loss). Electron density isosurfaces are displayed at an isovalue of $5{\cdot}10^{-5}$.

Single-point time-dependent density functional theory (TD-DFT) calculations (ORCA[21], PBE0, 6-31G(d)[22-24]) were undertaken for the single-layer Py-TT COF fragment displayed in Supplementary Figure 18a. This fragment was generated from the DFT/CASTEP-optimized and Rietveld-refined structure model of the Py-TT COF and terminated with hydrogen atoms at the 2-positions of the eight outer thienothiophene moieties.

The calculated orbital energies and occupancies in the ground state are as given in Supplementary Table 3.

Supplementary Table 3 | Selected ground state orbital energies.

Orbital	Occupancy	Energy (Eh)	Energy (eV)
829	2.000	-0.175018	-4.7625
830	2.000	-0.173696	-4.7265
831	0.000	-0.094949	-2.5837
832	0.000	-0.094862	-2.5813

In vacuo, the first excited state is mainly described by the following one-electron transitions:

829 → 831 (20.5 %)

830 → 832 (73.2 %)

For a qualitative analysis of the lowest-energy vertical electronic transition we focus on the difference in electron density on going from the ground state to the first excited state (Supplementary Figure 18d). The difference plot provides clear evidence of a charge-transfer from the electron-rich pyrene moieties to the thienothiophene bridges (orange = electron density gain, blue = loss), as we anticipated from our initial considerations and experiments.

We are aware that DFT can have difficulties to provide a quantitative description for excited states with strong charge-transfer character and for degenerate or pseudo-degenerate states.[25-27] A quantitative analysis using, e.g., double-hybrid functionals or even coupled-cluster methods, however, is beyond the scope of the present study.

In support of the experimental data, the solvent shift in water (ε_r = 80.4) is calculated by the conductor-like polarizable continuum model (CPCM)[28], and shows a positive solvatochromic effect on the ground state absorption.

Absorption (vacuum): 685 nm

Absorption (water): 705 nm

Supplementary References

1. Clark, S. J. *et al.* First principles methods using CASTEP. *Z. Kristallogr.* **220**, 567-570 (2005).
2. Perdew, J. P., Burke, K. & Ernzerhof, M. Generalized Gradient Approximation Made Simple. *Phys. Rev. Lett.* **77**, 3865-3868 (1996).
3. Monkhorst, H. J. & Pack, J. D. Special points for Brillonin-zone integrations. *Phys. Rev. B* **13**, 5188-5192 (1976).
4. Tkatchenko, A. & Scheffler, M. Accurate Molecular Van Der Waals Interactions from Ground-State Electron Density and Free-Atom Reference Data. *Phys. Rev. Lett.* **102**, 073005 (2009).
5. Düren, T., Millange, F., Férey, G., Walton, K. S. & Snurr, R. Q. Calculating Geometric Surface Areas as a Characterization Tool for Metal-Organic Frameworks. *J. Phys. Chem. C* **111**, 15350-15356 (2007).
6. www.fluidat.com. FLUIDAT on the Net, mass flow and physical properties calculations. (2007).
7. http://www.omega.com/temperature/z/pdf/z103.pdf. Equilibrium Relative Humidity Saturated Salt Solutions. (2000).
8. Auras, F. *et al.* Synchronized Offset Stacking: A Concept for Growing Large-Domain and Highly Crystalline 2D Covalent Organic Frameworks. *J. Am. Chem. Soc.* **138**, 16703-16710 (2016).
9. Jin, S. *et al.* Two-Dimensional Tetrathiafulvalene Covalent Organic Frameworks: Towards Latticed Conductive Organic Salts. *Chem. Eur. J.* **20**, 14608-14613 (2014).
10. Rabbani, M. G., Sekizkardes, A. K., El-Kadri, O. M., Kaafaranic, B. R. & El-Kaderi, H. M. Pyrene-directed growth of nanoporous benzimidazole-linked nanofibers and their application to selective CO2 capture and separation. *J. Mater. Chem.* **22**, 25409-25417 (2012).
11. Tountas, M. *et al.* Low Work Function Lacunary Polyoxometalates as Electron Transport Interlayers for Inverted Polymer Solar Cells of Improved Efficiency and Stability. *ACS Appl. Mater. Interfaces* **9**, 22773-22787 (2017).
12. Medina, D. D. *et al.* Oriented Thin Films of a Benzodithiophene Covalent Organic Framework. *ACS Nano* **8**, 4042-4052 (2014).
13. Bessinger, D., Ascherl, L., Auras, F. & Bein, T. Spectrally Switchable Photodetection with Near-Infrared-Absorbing Covalent Organic Frameworks. *J. Am. Chem. Soc.* **139**, 12035-12042 (2017).
14. Calik, M. *et al.* Extraction of Photogenerated Electrons and Holes from a Covalent Organic Framework Integrated Heterojunction. *J. Am. Chem. Soc.* **136**, 17802-17807 (2014).
15. Medina, D. D. *et al.* Directional Charge-Carrier Transport in Oriented Benzodithiophene Covalent Organic Framework Thin Films. *ACS Nano* **11**, 2706-2713 (2017).
16. Murgatroyd, P. N. Theory of space-charge-limited current enhanced by Frenkel effekt. *J. Phys. D: Appl. Phys.* **3**, 151-156 (1970).
17. Blom, P. W. M., de Jong, M. J. M. & Liedenbaum, C. T. H. F. Device Physics of Polymer Light-emitting Diodes. *Polym. Adv. Technol.* **9**, 390-401 (1998).
18. Kabra, D., Lu, L. P., Song, M. H., Snaith, H. J. & Friend, R. H. Effi cient Single-Layer Polymer Light-Emitting Diodes. *Adv. Mater.* **22**, 3194-3198 (2010).
19. Côte, A. P. *et al.* Porous, Crystalline, Covalent Organic Frameworks. *Science* **310**, 1166-1170 (2005).
20. Fang, Q. *et al.* Designed synthesis of large-pore crystalline polyimide covalent organic frameworks. *Nat. Commun.* **5**, 4503 (2014).
21. Neese, F. The ORCA program system. *WIREs Comput. Mol. Sci.* **2**, 73-78 (2012).
22. Ditchfield, R., Hehre, W. J. & Pople, J. A. Self-Consistent Molecular-Orbital Methods. IX. An Extended Gaussian-Type Basis for Molecular-Orbital Studies of Organic Molecules. *J. Chem. Phys.* **54**, 724-728 (1971).
23. Hehre, W. J., Ditchfield, R. & Pople, J. A. Self-Consistent Molecular Orbital Methods. XII. Further Extensions of Gaussian-Type Basis Sets for Use in Molecular Orbital Studies of Organic Molecules. *J. Chem. Phys.* **56**, 2257-2261 (1972).
24. Francl, M. M. *et al.* Self-consistent molecular orbital methods. XXIII. A polarization-type basis set for second-row elements. *J. Chem. Phys.* **77**, 3654-3665 (1982).
25. Kaplan, I. G. Problems in DFT with the Total Spin and Degenerate States. *Int. J. Quant. Chem.* **107**, 2595-2603 (2007).
26. Grimme, S. & Neese, F. Double-hybrid density functional theory for excited electronic states of molecules. *J. Chem. Phys.* **127**, 154116 (2007).

27 Goerigk, L. & Grimme, S. A thorough benchmark of density functional methods for general main group thermochemistry, kinetics, and noncovalent interactions. *Phys. Chem. Chem. Phys.* **13**, 6670-6688 (2011).

28 Cossi, M., Rega, N., Scalmani, G. & Barone, V. Energies, structures, and electronic properties of molecules in solution with the C-PCM solvation model. *J. Comput. Chem.* **24**, 669-681 (2003).
