# Supplementary material for: Solvatochromic covalent organic frameworks
Source: Nat Commun. 2018 Sep 18;9:3802. doi: 10.1038/s41467-018-06161-w (PMC6143592; doi:10.1038/s41467-018-06161-w)
Supplement: Supplementary file 2 — Description of Additional Supplementary Files [file 41467_2018_6161_MOESM2_ESM.pdf]

Description of Additional Supplementary Files

Supplementary Movie 1

Description: Demonstration of the COF-based humidity sensor. The light emitted from the green LED (positioned in the back) is transmitted through the Py-TT COF film (orange or red, depending on the respective atmosphere) and detected via a light-dependent resistor (front). In a dry atmosphere, the COF is almost transparent to green light, whereas it becomes increasingly opaque with increasing humidity.

Supplementary Data 1

Description: Rietveld-refined structure model of the Py-TT COF. The COF geometry is optimized by DFT using the experimentally determined unit cell parameters (for details see the Supplementary Methods).

Supplementary Data 2

Description: Rietveld-refined structure model of the Py-1P COF. The COF geometry is optimized by force-field methods using the experimentally determined unit cell parameters (for details see the Supplementary Methods).

Supplementary Data 3

Description: Rietveld-refined structure model of the Py-Py COF. The COF geometry is optimized by force-field methods using the experimentally determined unit cell parameters (for details see the Supplementary Methods).
